# Arabidopsis TRB Proteins Form Two Closely Related Complexes to Mediate H3K4me3 Demethylation and Transcriptional Repression

**DOI:** 10.1002/advs.202503420

**Published:** 2025-10-05

**Authors:** Qi Wang, Dan‐Yang Yuan, Yu‐Jia Lu, Xin Wang, Hao Zheng, Lin Li, She Chen, Xin‐Jian He

**Affiliations:** ^1^ College of Life Sciences Beijing Normal University Beijing 100875 China; ^2^ National Institute of Biological Sciences Beijing 102206 China; ^3^ Tsinghua Institute of Multidisciplinary Biomedical Research Tsinghua University Beijing 10084 China

**Keywords:** H3K4me3, histone demethylation, JMJ14, transcriptional repression, TRB1

## Abstract

The Arabidopsis telomere repeat binding proteins TRB1, TRB2, and TRB3 (TRB1/2/3) are components of the PWWP‐EPCR‐ARID‐TRB (PEAT) complex, which is involved in histone acetylation and H2A deubiquitination. TRB1/2/3 also interact with the Polycomb Repressive Complex 2 (PRC2), which mediates H3 lysine 27 trimethylation. This study demonstrates that TRB1/2/3 can form two closely related complexes: TRB1/2/3‐HELIX‐TURN‐HELIX‐PROTEIN (TRHT) and TRB1/2/3‐HISTONE‐DEMETHYLASE (TRHD). The TRHT and TRHD (TRHT/TRHD) complexes occupy numerous common target genes across the genome and are required for H3K4me3 demethylation mediated by the histone demethylase JMJ14. Within the TRHT/TRHD complexes, the transcriptional repressors TRB1/2/3 and NAC050/052 bind to distinct DNA motifs and cooperate to mediate the association of these complexes with their target genes. Compared to PEAT target genes with high H3K4me3 levels and PRC2 target genes with low H3K4me3 levels, TRHT/TRHD target genes display low‐to‐medium levels of H3K4me3. At TRHT/TRHD target genes, JMJ14‐mediated H3K4me3 demethylation is enhanced when these genes are shared with PRC2 target genes and is suppressed when they are shared with PEAT target genes. These findings reveal how the newly identified TRHT/TRHD complexes cooperate with the PEAT and PRC2 complexes to regulate multiple histone modifications and gene transcription across the genome.

## Graphical abstract

This study reveals that Arabidopsis telomere repeat binding proteins (TRBs) form two closely related complexes. These complexes mediate H3K4me3 demethylation and transcriptional repression through a positive feedback loop and cooperate with PEAT and PRC2 complexes to regulate multiple histone modifications and gene expression across the genome.

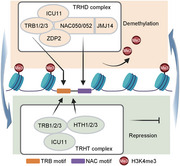

## 1 Introduction

In the chromatin of eukaryotes, the N‐terminal tails of histone protrude from the nucleosome core and undergo diverse covalent post‐translational modifications (PTMs). These modifications, such as acetylation, methylation, ubiquitination, and phosphorylation, are pivotal for the regulation of transcription, DNA replication, and DNA damage repair.^[^ ^1^, ^2^, ^3^, ^4^ ^]^ Among these modifications, histone 3 lysine 4 trimethylation (H3K4me3) is associated with active transcription, whereas histone 3 lysine 27 trimethylation (H3K27me3) is linked to transcriptional repression.^[^ ^5^, ^6^, ^7^ ^]^ Trithorax and polycomb histone methyltransferases deposit H3K4me3 and H3K27me3, respectively.^[^ ^8^, ^9^, ^10^, ^11^, ^12^, ^13^, ^14^ ^]^ Specifically, polycomb histone methyltransferases typically form Polycomb Repressive Complex 2 (PRC2) and function as the catalytic subunits of this complex.^[^ ^15^, ^16^ ^]^ INCURVATA 11 (ICU11) was reported to function as an accessory subunit of PRC2, thereby mediating H3K27 trimethylation and transcriptional repression during vernalization at a key flowering repressor gene, *FLOWERING LOCUS C* (*FLC*).^[^ ^17^ ^]^

Histone acetylation occurs at multiple lysine sites on H3 and H4 and is consistently associated with active transcription.^[^ ^18^, ^19^, ^20^ ^]^ In *Arabidopsis thaliana*, two redundant MYST‐type histone acetyltransferases, HAM1 and HAM2 (HAM1/2), catalyze H4 lysine 5 acetylation (H4K5ac) and are essential for plant viability.^[^ ^21^, ^22^ ^]^ HAM1/2 are shared catalytic subunits of the conserved NuA4 histone acetyltransferase complex and the plant‐specific PWWP‐EPCR‐ARID‐TRB (PEAT) complex.^[^ ^23^, ^24^, ^25^, ^26^, ^27^ ^]^ The PEAT complex also contains UBP5, a deubiquitinase of H2Aub, thus coupling histone acetylation with H2A deubiquitination.^[^ ^25^, ^28^ ^]^ Although numerous histone modifications have been identified, the mechanisms by which these modifications are regulated in a locus‐dependent manner and how they are coordinated at the whole‐genome level remain to be explored.

The methyl groups on H3K4me3 and H3K27me3 can be actively removed by histone lysine demethylases.^[^ ^29^, ^30^, ^31^ ^]^ Histone lysine demethylases are classified into two types: flavin adenine dinucleotide (FAD)‐dependent LSD/KDM1 family demethylases and α‐ketoglutarate‐ and Fe^2+^‐dependent Jumonji domain‐containing demethylases.^[^ ^32^, ^33^ ^]^ In Arabidopsis, FLD, a well‐characterized FAD‐dependent histone demethylase, mediates H3K4me1/2 demethylation at the key flowering repressor gene *FLC*, thereby promoting flowering through an autonomous pathway.^[^ ^34^, ^35^, ^36^ ^]^ Two additional Arabidopsis LSD‐like proteins LDL1 and LDL2 are redundantly involved in the regulation of flowering time.^[^ ^32^ ^]^ JUMONJI14 (JMJ14) is a Jumonji domain‐containing histone demethylase that mediates histone demethylation at the lysine 4 site of H3, and is involved in the regulation of flowering time and RNA‐directed DNA methylation.^[^ ^37^, ^38^, ^39^, ^40^ ^]^ REF6 is a Jumonji domain‐containing histone demethylase that specifically removes the methyl group from H3K27me3.^[^ ^41^ ^]^ REF6 is recruited to chromatin through the sequence‐dependent binding ability of its zinc‐finger domain.^[^ ^42^, ^43^ ^]^ Unlike REF6, JMJ14 is recruited to its target genomic loci by interacting with NAC050 and NAC052 (NAC050/052), two redundant transcriptional repressors, thereby mediating H3K4me3 demethylation and transcriptional repression in a locus‐dependent manner.^[^ ^38^, ^44^ ^]^

TELOMERE REPEAT BINDING PROTEIN 1 (TRB1), TRB2, and TRB3 (TRB1/2/3), initially identified as telomere‐interacting proteins,^[^ ^45^ ^]^ were later shown to interact with components of the PRC2 complex, contributing to H3K27 trimethylation and transcriptional repression.^[^ ^46^ ^]^ TRB1/2 were also found to be components of the PEAT complex, which is involved in histone acetylation and H2A deubiquitination.^[^ ^24^, ^25^ ^]^ Although components of the PRC2 and PEAT complexes are crucial for plant development, loss‐of‐function mutants of core PRC2 and PEAT components display severe developmental defects but are not lethal.^[^ ^24^, ^47^, ^48^ ^]^ In contrast, while the *trb1* and *trb3* single mutants did not exhibit visible phenotypes compared to the wild type,^[^ ^24^, ^46^ ^]^ a homozygous *trb2* mutant was not detected in the progeny of the self‐fed heterozygous *trb2* mutant,^[^ ^24^ ^]^ indicating that TRB2 is likely essential for plant viability. This suggests that TRB1/2/3 are more significant than the canonical components of the PRC2 and PEAT complexes in regulating plant development. Additionally, TRB1 and TRB INTERACTING PROTEIN 1 (TRBIP1) are required for recruiting JMJ14 to target genomic loci, thereby facilitating H3K4me3 demethylation and DNA methylation.^[^ ^39^, ^49^ ^]^ However, unlike TRB1/2/3, which are essential for plant development,^[^ ^24^, ^46^ ^]^ JMJ14 and NAC050/052 are specifically involved in the regulation of flowering time.^[^ ^38^, ^44^ ^]^ These results suggest that TRB1/2/3 are involved in regulating various developmental processes by interacting with multiple histone modifiers. However, it remains largely unclear how TRB1/2/3 contribute to the regulation of histone modifications and gene transcription at the whole‐genome level.

In this study, we discovered that, in addition to the previously defined TRB‐interacting PEAT and PRC2 complexes,^[^ ^24^, ^25^ ^]^ TRB1/2/3 can form two closely related chromatin‐associated complexes: TRB1/2/3‐HELIX‐TURN‐HELIX‐PROTEIN (TRHT) and TRB1/2/3‐HISTONE DEMETHYLASE (TRHD). In the TRHT complex, TRB1/2/3 interact with INCURVATA 11 (ICU11) and three redundant homologs, HELIX‐TURN‐HELIX PROTEIN 1, 2, and 3 (HTH1, 2, and 3), in which HTH2 was recently termed as TRBIP1.^[^ ^49^ ^]^ In the TRHD complex, TRB1/2/3 interact with ICU11, Z INC‐FINGER‐DOMAIN PROTEIN 2 (ZDP2), the H3K4me3 demethylase JUMONJI 14 (JMJ14), and the NAC transcriptional repressors, NAC050 and NAC052. We found that the Myb domain of TRB1 exhibits a sequence‐dependent DNA‐binding ability and is involved in the association of TRHT and TRHD complexes with chromatin. Compared with the PEAT and PRC2 target genes, which are characterized by high and low levels of H3K4me3, respectively, the TRHT and TRHD (TRHT/TRHD) complexes occupy a common set of genes with low‐to‐medium levels of H3K4me3. At the TRHT/TRHD target genes, the TRHT/TRHD complexes mediate transcriptional repression through both H3K4me3 demethylation‐dependent and ‐independent mechanisms, and the TRHT/TRHD‐dependent H3K4me3 demethylation is enhanced at PRC2 target genes and is suppressed at PEAT target genes. These findings elucidate how the TRHT/TRHD, PEAT, and PRC2 complexes are coordinated to regulate histone modifications and gene transcription at the whole‐genome level, highlighting the critical role of these complexes in orchestrating various developmental processes.

## 2 Results

### 2.1 Identification of TRB1/2/3‐Containing TRHT and TRHD Complexes

**Figure 1:**
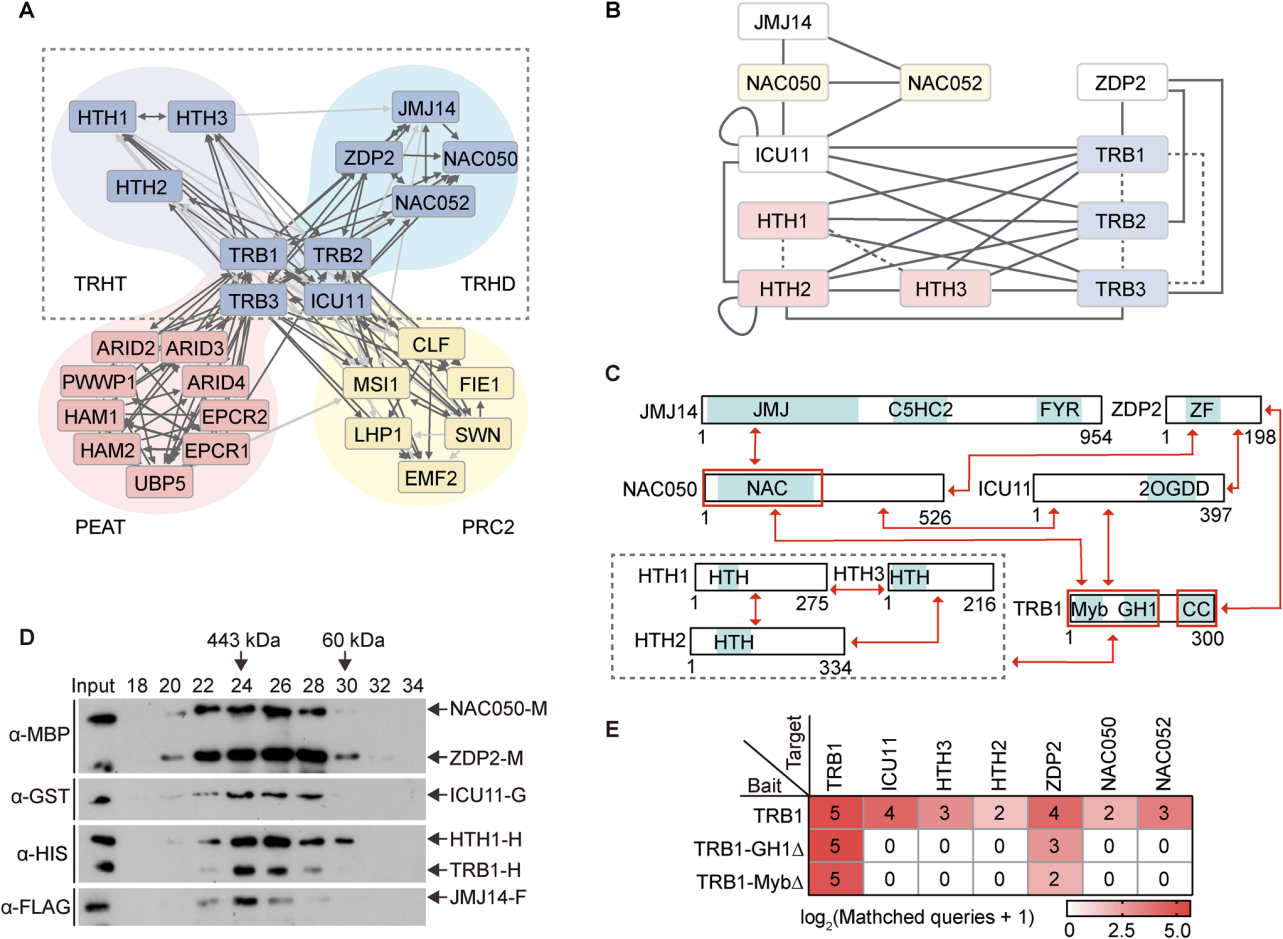
Identification and characterization of TRHT and TRHD complexes in Arabidopsis. A) Diagram indicating the interactions among components of the TRHT, TRHD, PEAT, and PRC2 complexes, as determined by AP‐MS. The diagram was generated using Cytoscape (v3.6.1) based on the AP‐MS data. Arrows indicate proteins co‐purified with the baits. Light gray lines represent the log_2_(matched queries + 1) value of AP‐MS less than 2, while dark gray lines represent the log_2_(matched queries + 1) value of AP‐MS greater than 2. B) Schematic representation of protein‐protein interactions determined by the yeast two‐hybrid assay. Gray dash and gray solid lines denote interactions between the indicated proteins at 0 or 3 mm 3‐amino‐1,2,4‐triazole (3‐AT) SD‐LWH. C) Schematic representation of protein‐protein interactions determined by pull‐down assays. Bidirectional red arrows represent interactions between the indicated proteins. The dashed gray box encloses homologous proteins. Red boxes highlight interaction fragments. JMJ, jumonji domain; C5HC2, zinc‐finger C5HC2‐type domain; FYR, FY‐rich domain; NAC, NAM/ATAF1/2/CUC domain; 2OGDD, 2‐oxoglutarate‐dependent dioxygenase domain; HTH, helix‐turn‐helix domain; GH1, globular histone 1 domain; CC, coiled‐coil domain; ZF, zinc‐finger domain. D) Determination of TRHT/TRHD formation in vitro. Individual proteins purified from *E. coli* were mixed in equivalent molar amounts and separated in a Superose 6 column, and detected by immunoblotting. M, MBP. G, GST. H, HIS. F, FLAG. E) Proteins co‐purified with wild‐type TRB1, TRB1‐GH1∆, and TRB1‐Myb∆ proteins, as determined by AP‐MS in Arabidopsis. Transgenic plants expressing Flag‐tagged wild‐type TRB1, TRB1‐GH1∆, and TRB1‐Myb∆ proteins were employed in AP‐MS. The abundance of purified proteins is represented by the identified queries detected via AP‐MS.

Our previous affinity purification coupled with mass spectrometry (AP‐MS) analysis identified TRB1 and TRB2 as redundant components of PWWP‐EPCR‐ARID‐TRB (PEAT) complex, which is involved in histone acetylation and H2A deubiquitination.^[^ ^24^, ^25^ ^]^ Additional studies have demonstrated that TRB1, TRB2, and TRB3 (TRB1/2/3) can interact with components of the Polycomb Repressive Complex 2 (PRC2) to mediate deposition of H3K27 trimethylation.^[^ ^46^, ^50^ ^]^ Furthermore, they interacted with the H3K4me3 demethylase JUMONJI 14 (JMJ14) and its associated NAC transcriptional repressors, NAC050/052.^[^ ^38^, ^39^, ^44^ ^]^ To further investigate the complexes comprising TRB1/2/3, we conducted a systematic analysis of proteins co‐purified with TRB1/2/3 based on AP‐MS analysis.^[^ ^24^, ^25^, ^39^ ^]^ This investigation revealed four previously uncharacterized TRB1/2/3‐interacting proteins. One of these proteins contains a zinc finger domain and is designated ZINC‐FINGER‐DOMAIN PROTEIN 2 (ZDP2), while the other three are homologous proteins featuring a conserved HELIX‐TURN‐HELIX (HTH) domain, referred to as HTH1, HTH2, and HTH3 (**Figure** **1**A; Figure S1 and Dataset S1, Supporting Information). Our nuclear‐cytoplasmic fractionation assays indicated that TRB1, ZDP2, and HTH1 were exclusively located in the nucleus, while ICU11, JMJ14, and NAC052 were present in both the nucleus and cytoplasm (Figure S2, Supporting Information). These results suggest that TRB1/2/3 and their potential interacting proteins are primarily located in the nucleus.

To verify the interactions between TRB1/2/3 and their potential interacting proteins, we generated transgenic plants expressing these proteins tagged with the Flag epitope for AP‐MS analysis. The AP‐MS data indicated that TRB1/2/3 were also co‐purified with these proteins (Figure 1A; Figure S1 and Dataset S1, Supporting Information), thereby validating their interactions. Additionally, a combined analysis of the AP‐MS results enabled the identification of two previously uncharacterized TRB1/2/3‐containing complexes, in addition to the known PEAT and PRC2 complexes (Figure 1A). One of the newly identified complexes, termed TRB1/2/3‐HELIX‐TURN‐HELIX‐PROTEIN (TRHT), consists of TRB1/2/3, ICU11, and HTH1/2/3, whereas the other, named TRB1/2/3‐HISTONE‐DEMETHYLASE (TRHD), includes TRB1/2/3, ICU11, JMJ14, NAC050/052, and ZDP2 (Figure 1A). In these components, TRB1/2/3 are shared by the TRHT, TRHD, PEAT, and PRC2 complexes, while ICU11 is shared by the TRHT, TRHD, and PRC2 complexes. HTH1/2/3 are unique to the TRHT complex, and JMJ14, NAC050/052, and ZDP2 are unique to the TRHD complex (Figure 1A).

We conducted yeast two‐hybrid assays to explore the interactions among the components of the TRHT and TRHD complexes. Previous studies from our group and others have shown the interactions between JMJ14 and NAC050/052,^[^ ^38^, ^44^ ^]^ and this finding was supported by our current yeast two‐hybrid results (Figure S3, Supporting Information). Additionally, we found that all components of the TRHT and TRHD complexes, except for JMJ14 and NAC050/052, can interact with TRB1/2/3, reinforcing the concept that TRB1/2/3 serve as shared subunits of both complexes. Within the TRHD complex, JMJ14 specifically interacts with NAC050/052 and not with any other components (Figure 1B; Figure S3, Supporting Information), indicating that JMJ14 is recruited to the complex solely through its interaction with NAC050/052. Notably, the yeast two‐hybrid assays did not detect any interactions between the TRHT‐specific subunits and the TRHD‐specific subunits (Figure 1B; Figure S3, Supporting Information), further reinforcing the notion that the TRHT complex is distinct from the TRHD complex.

Furthermore, we performed in vitro pull‐down assays to investigate the interactions among the components of the TRHT and TRHD complexes. Consistent with the yeast two‐hybrid results (Figure 1B), the pull‐down assays did not detect any interactions between the TRHT‐specific components and the TRHD‐specific components (Figure 1C; Figure S4A,B, Supporting Information). The pull‐down assays confirmed the interaction between TRB1 and ICU11, two shared subunits of the TRHT and TRHD complexes (Figure 1C). In the TRHT complex, TRB1 interacts with HTH1/2/3, while ICU11 does not (Figure 1C). In the TRHD complex, both TRB1 and ICU11 interact with ZDP2 and NAC050, but neither interacts with JMJ14 (Figure 1C), further substantiating the idea that JMJ14 is recruited to the TRHD complex through its interaction with NAC050/052. Additionally, the pull‐down assays revealed an interaction between ZDP2 and NAC050, two TRHD‐specific subunits (Figure 1C). Gel filtration assays demonstrated that TRHT/TRHD components formed high‐molecular‐weight complexes in vitro (Figure 1D), suggesting the formation of stable TRHT/TRHD complexes.

Using truncated versions of TRB1 (Figure S5, Supporting Information), the pull‐down assays indicated that TRB1 interacts with ZDP2 through its C‐terminal coiled‐coil domain and engages with other TRHT or TRHD components through its N‐terminal region, which contains both the Myb and GH1 domains (Figure 1C). The distinct interaction modes were further demonstrated by AP‐MS using transgenic plants expressing Myb‐deleted TRB1 (TRB1‐MybΔ) and GH1‐deleted TRB1 (TRB1‐GH1Δ) (Figure 1E). These results confirm the formation of two distinct TRB1/2/3‐containing complexes: TRHT and TRHD.

### 2.2 TRHT and TRHD Complexes Cooperate to Regulate Plant Development and Gene Expression

**Figure 2:**
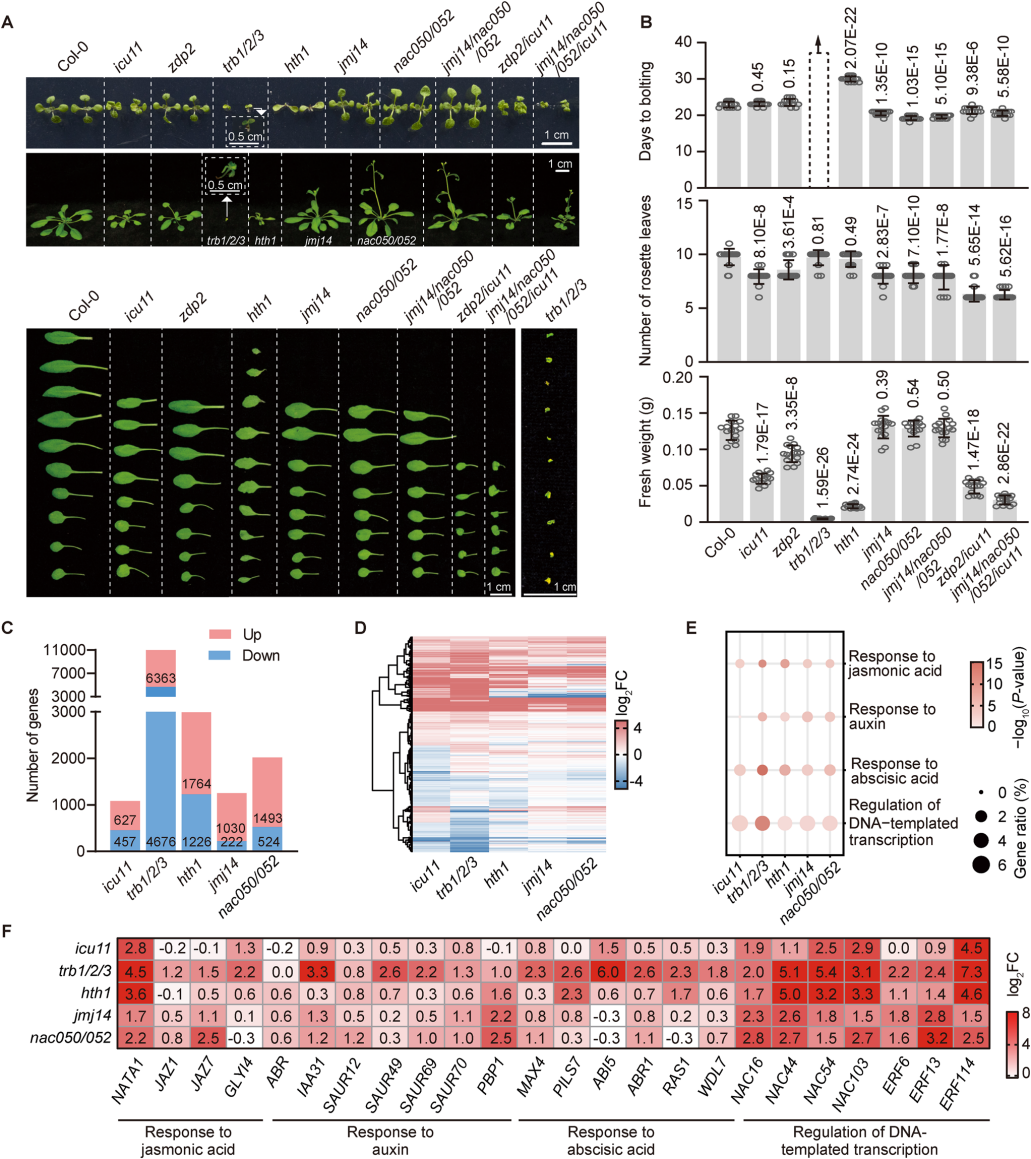
The TRHT and TRHD complexes contribute to plant growth and development by regulating gene expression. A) Morphological phenotypes of TRHT and TRHD mutants. Shown are the phenotypes of 12‐day‐old plants (top), 23‐day‐old plants (middle), and leaves excised from 26‐day‐old plants (bottom). B) Statistical results of days to bolting (top), number of rosette leaves in 30‐day‐old plants (middle), and fresh weight of 26‐day‐old plants (bottom). Flowering time was assessed as the number of days to bolting. Mean values and standard deviation (SD) are calculated from at least 16 plants. *P* values were determined by the two‐tailed Student's t‐test. C) The number of up‐ and down‐regulated DEGs as determined by RNA‐seq in the indicated mutants relative to the wild type. D) Heatmaps showing gene expression changes in indicated mutants relative to the wild type according to DEGs in *icu11*. Red and blue bars represent the expression changes of up‐ and down‐regulated DEGs, respectively. FC, fold change. E) Gene ontology analysis of the upregulated DEGs identified by RNA‐seq in the indicated mutants relative to the wild type. F) The expression change of representative upregulated DEGs in the indicated mutants relative to the wild type. Red represents the gene expression change (log_2_FC).

To investigate the biological functions of the TRHT and TRHD complexes, we aimed to obtain mutants for all components of these complexes and examine their developmental phenotypes. As previously reported,^[^ ^24^ ^]^ the homozygous loss‐of‐function *trb2‐2* (GABI_103E02) single mutant was absent from the progeny of its self‐fed heterozygous mutant (Figure S6A,B, Supporting Information), suggesting that TRB2 is essential for plant development. We generated *trb2* and *trb3* mutations using CRISPR‐Cas9‐induced mutagenesis, and obtained a viable *trb1 trb2 trb3* (*trb1/2/3*) triple mutant that contains a weak *trb2* mutant allele (Figure S6C,D, Supporting Information). While the single and double *trb* mutants did not display any visible developmental defects, the *trb1/2/3* triple mutant exhibited severe developmental abnormalities, including a reduction in plant size and a failure to bolt (**Figure** **2**A,B; Figure S7A,B, Supporting Information). These findings are consistent with those reported in a previously characterized *trb1/2/3* mutant.^[^ ^46^ ^]^ We generated *hth1*, *hth2, hth3*, *icu11*, and *zdp2* mutations using CRISPR‐Cas9‐induced mutagenesis (Figure S6E, Supporting Information). Notable phenotypes were observed in *hth1*, *icu11*, and *zdp2* mutants, but not in *hth2* or *hth3* mutants (Figure 2A; Figure S7C,D, Supporting Information). The development phenotypes of these mutants were confirmed by complementation testing (Figure S7E,F, Supporting Information). Moreover, the *hth1/hth3* double mutant did not exhibit more severe phenotypes than the *hth1* single mutant, and the *hth2/3* double mutant had no visible phenotypes (Figure S7C,D, Supporting Information). Consistent with previous reports,^[^ ^38^, ^44^ ^]^ the *jmj14* and *nac050/nac052* mutants exhibited early flowering and a reduced number of rosette leaves upon flowering compared to the wild type (Figure 2A,B).

To explore the relationships between different components of the TRHD complex, we combined mutations in these components and analyzed their effects. First, we created a *jmj14/nac050/052* triple mutant, and demonstrated the early flowering of this mutant (Figure 2A,B). We then introduced the *icu11* mutation into the *jmj14/nac050/052* triple mutant, thereby obtaining *jmj14/nac050/052/icu11* quadruple mutant. Compared to either the *icu11* single mutant or the *jmj14/nac050/052* triple mutant, the quadruple mutant showed reduced plant weight and fewer rosette leaves, while its flowering time was not further accelerated relative to the *jmj14/nac050/052* triple mutant (Figure 2A,B). Additionally, we combined the *zdp2* and *icu11* mutations to produce a *zdp2/icu11* double mutant. Notably, this double mutant exhibited early flowering and reduced number of rosette leaves, compared to the wild type (Figure 2A,B), suggesting that ZDP2 or ICU11 can function synergistically to modulate flowering time. This supports the role of the TRHD complex in regulating flowering time. Together, these results suggest that distinct components of the TRHD complex cooperate to regulate multiple developmental processes.

We conducted RNA deep sequencing (RNA‐seq) analysis to investigate how the TRHT and TRHD complexes cooperate to regulate gene expression at the whole‐genome level. Three biological replicates of each genotype confirmed the high quality of the RNA‐seq data. We identified numerous differentially expressed genes (DEGs) (log_2_ [fold change] > 0.5 or < −0.5, FDR < 0.05) in each mutant compared to the wild type. The number of upregulated DEGs consistently exceeded that of down‐regulated ones in each mutant (Figure 2C,D; Dataset S2, Supporting Information), supporting the notion that the TRHT and TRHD complexes function as transcriptional repressors. Gene Ontology (GO) analysis revealed that the up‐regulated DEGs in the TRHT and TRHD mutants were enriched in biological processes such as DNA binding, transcription, stress responses, and protein ubiquitination (Figure 2E; Dataset S3, Supporting Information). Notably, many key genes associated with these processes were significantly elevated in all TRHT and TRHD mutants (Figure 2F), indicating that they can co‐regulate gene expression in these pathways.

### 2.3 TRHT and TRHD Complexes Bind Common Target Genomic Loci

**Figure 3:**
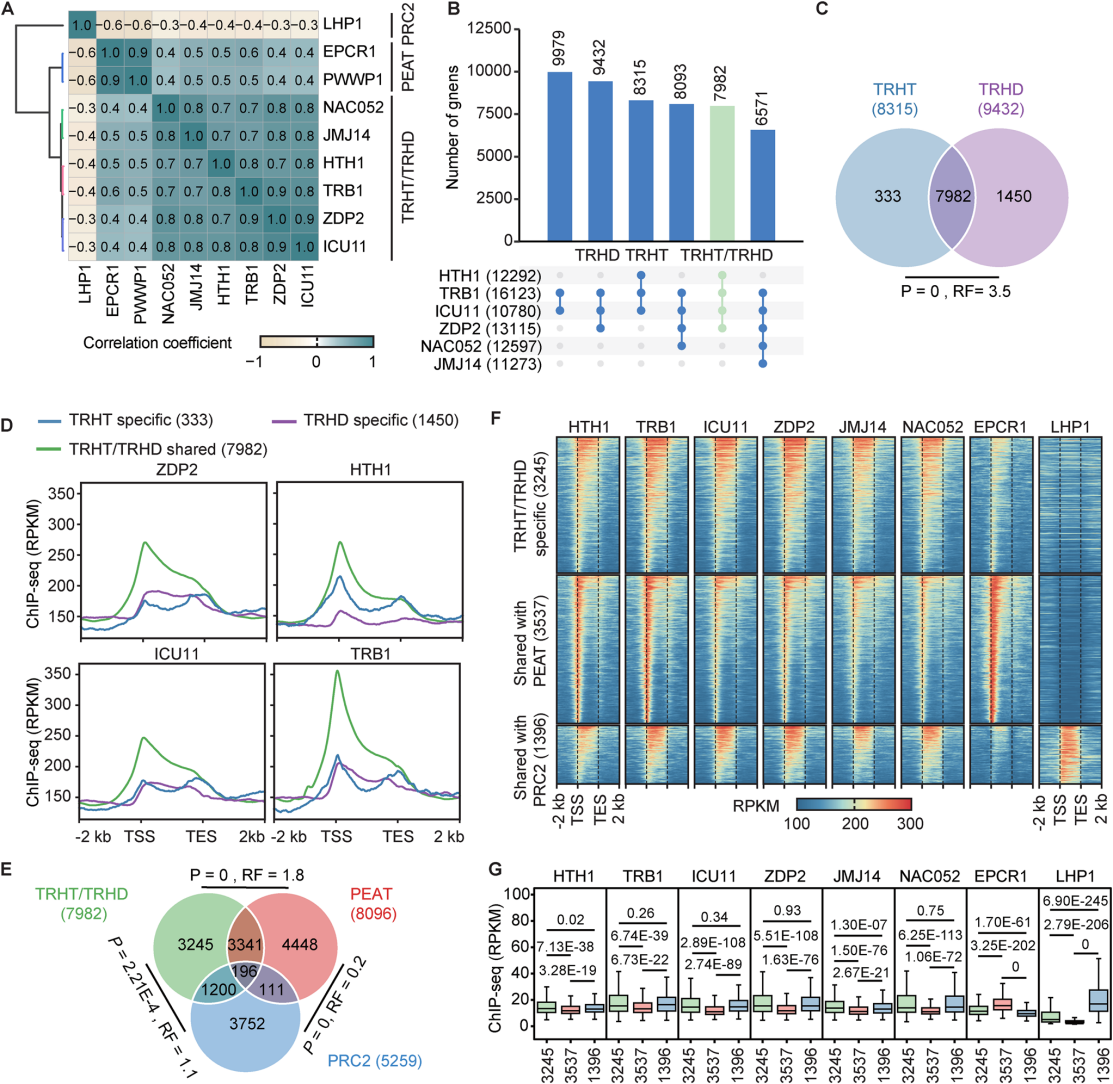
Comparison of genomic regions bound by TRHT/TRHD, PEAT, and PRC2 complexes. A) Correlation analysis of ChIP‐seq signals among TRHT/TRHD, PEAT, and PRC2 components. Pearson correlation coefficients are shown. B) Upset plot showing the number of genes bound by individual TRHT/TRHD components and their overlapping gene sets. Genes co‐bound by TRB1, ICU11, and ZDP2 represent TRHD targets (9432). Genes co‐bound by TRB1, ICU11, and HTH1 represent TRHT targets (8315). Genes shared by both TRHT and TRHD are referred to as TRHT/TRHD common targets (7982). C) Venn diagram showing the overlap between TRHT and TRHD target genes. The three subclasses of TRHT/TRHD target genes include TRHT‐specific (333), TRHD‐specific (1450), and TRHT/TRHD‐shared (7982) targets. *P* values were calculated using the one‐tailed hypergeometric test. RF represents the ratio of observed to expected overlapping genes. D) Meta plots showing differential distribution of TRHT and TRHD ChIP‐seq signals over their target genes. TSS, transcription start site; TES, transcription end site. E) Venn diagram showing the overlap among TRHT/TRHD, PEAT, and PRC2 target genes. *P* values were calculated using the two‐tailed hypergeometric test. RF represents the ratio of observed to expected overlapping genes. F) Heatmaps displaying the ChIP‐seq enrichment of ICU11, JMJ14, HTH1, TRB1, EPCR1, and LHP1 at three subclasses of TRHT/TRHD target genes: TRHT/TRHD‐specific (3245), TRHT/TRHD‐ and PEAT‐shared (3537), and TRHT/TRHD‐ and PRC2‐shared (1396). TSS, transcription start site; TES, transcription end site. G) Boxplots showing ChIP‐seq enrichment levels of indicated factors at the three subclasses of TRHT/TRHD target genes. *P* values were determined by the two‐tailed Mann‐Whitney U test (unpaired).

To investigate whether the TRHT and TRHD complexes directly regulate gene expression at the chromatin level, we performed chromatin immunoprecipitation sequencing (ChIP‐seq) in transgenic plants expressing Flag‐tagged components of TRHT and TRHD complexes, including HTH1, TRB1, NAC052, JMJ14, ZDP2, and ICU11. A comparison of our TRB1 ChIP‐seq results with published TRB1 ChIP‐seq data^[^ ^46^, ^50^ ^]^ revealed a significant degree of concordance, confirming the reliability of our ChIP‐seq data (Figure S8A,B, Supporting Information). Correlation analysis indicated that the ChIP‐seq signals of the TRHT and TRHD (TRHT/TRHD) components were highly correlated (**Figure** **3**A), supporting the idea that these components are associated with common target genes.

Given that TRB1 and ICU11 are also components of the PEAT and/or PRC2 complexes,^[^ ^17^, ^24^, ^25^, ^46^ ^]^ we integrated the ChIP‐seq results from the PRC2 component LHP1 and the PEAT components EPCR1 and PWWP1 into our correlation analysis. This analysis demonstrated that the ChIP‐seq signals of the TRHT/TRHD components exhibited a positive correlation with those of the PEAT components, while showing a negative correlation with those of the PRC2 component (Figure 3A). The correlation among the TRHT/TRHD components was substantially higher than the correlation between the TRHT/TRHD components and the PEAT components (Figure 3A; Figure S8C, Supporting Information), suggesting that the TRHT/TRHD components bind to a specific set of genes. Notably, although TRB1 and ICU11 have been identified as components of the PEAT and/or PRC2 complexes,^[^ ^17^, ^24^, ^25^, ^46^ ^]^ the ChIP‐seq signals of TRB1 and ICU11 were more closely correlated with those of the TRHT/TRHD components than with those of the PEAT components, and were even negatively correlated with those of the PRC2 component (Figure 3A). This indicates that TRB1 and ICU11 play a more critical role in the TRHT/TRHD complexes than in the PEAT or PRC2 complexes.

To compare TRHT target genes and TRHD target genes, we defined 8315 genes co‐bound by TRB1, ICU11, and HTH1 as TRHT target genes, and 9432 genes co‐bound by TRB1, ICU11, and ZDP2 as TRHD target genes (Figure 3B; Dataset S4, Supporting Information). A Venn diagram revealed a significant overlap between TRHT and TRHD target genes (Figure 3C). Additionally, the enrichment levels of TRHT/TRHD components were markedly higher at target genes shared by both complexes than at those specific to either TRHT or TRHD (Figure 3D), indicating that TRHT and TRHD primarily co‐occupy chromatin on a genome‐wide scale.

### 2.4 Histone Modifications of TRHT/TRHD, PEAT, and PRC2 Target Genes

To characterize the target genes of TRHT/TRHD complexes, we compared the 7982 TRHT/TRHD target genes with the 8096 PEAT target genes (defined by genes co‐bound by the PEAT components EPCR2, UBP5, and PWWP1)^[^ ^25^ ^]^ and the 5259 PRC2 target genes (defined by genes bound by the PRC2 component LHP1). Venn diagram analysis revealed that the overlap between PEAT and PRC2 target genes was significantly lower than expected by chance (*P* = 0, RF = 0.2), indicating PEAT and PRC2 target entirely distinct genes. In contrast, the TRHT/TRHD target genes showed a substantial overlap with PEAT target genes (*P* = 0; RF = 1.8) and a relatively lower overlap with PRC2 target genes (*P* = 2.21E‐4, RF = 1.1) (Figure 3E). These findings confirm that the TRHT/TRHD target genes are distinct from both the PEAT and PRC2 target genes.

We categorized the TRHT/TRHD target genes into three groups: TRHT/TRHD‐specific target genes (3245), target genes shared with the PEAT complex (3537), and target genes shared with the PRC2 complex (1396). We then analyzed histone modification (H3K4me3, H3K27me3, H4K5ac, and H2Aub) and transcript levels of these genes in the wild type. Our analysis indicated that, compared to the TRHT/TRHD‐specific target genes, the target genes shared with the PEAT complex exhibited higher levels of H3K4me3 and H4K5ac, lower levels of H3K27me3 and H2Aub, and higher transcript levels. In contrast, the target genes shared with the PRC2 complex showed lower levels of H3K4me3, higher levels of H3K27me3 and H2Aub, and lower transcript levels (Figure 3F,G; Figure S8D,E, Supporting Information). These findings are consistent with previous reports that the PEAT and PRC2 complexes associate with active and repressive chromatin regions, respectively,^[^ ^25^, ^51^, ^52^, ^53^ ^]^ and indicate that TRHT/TRHD target genes exhibit an intermediate chromatin state between the PEAT and PRC2 target genes.

### 2.5 The TRHT and TRHD Complexes Mediate H3K4me3 Demethylation

**Figure 4:**
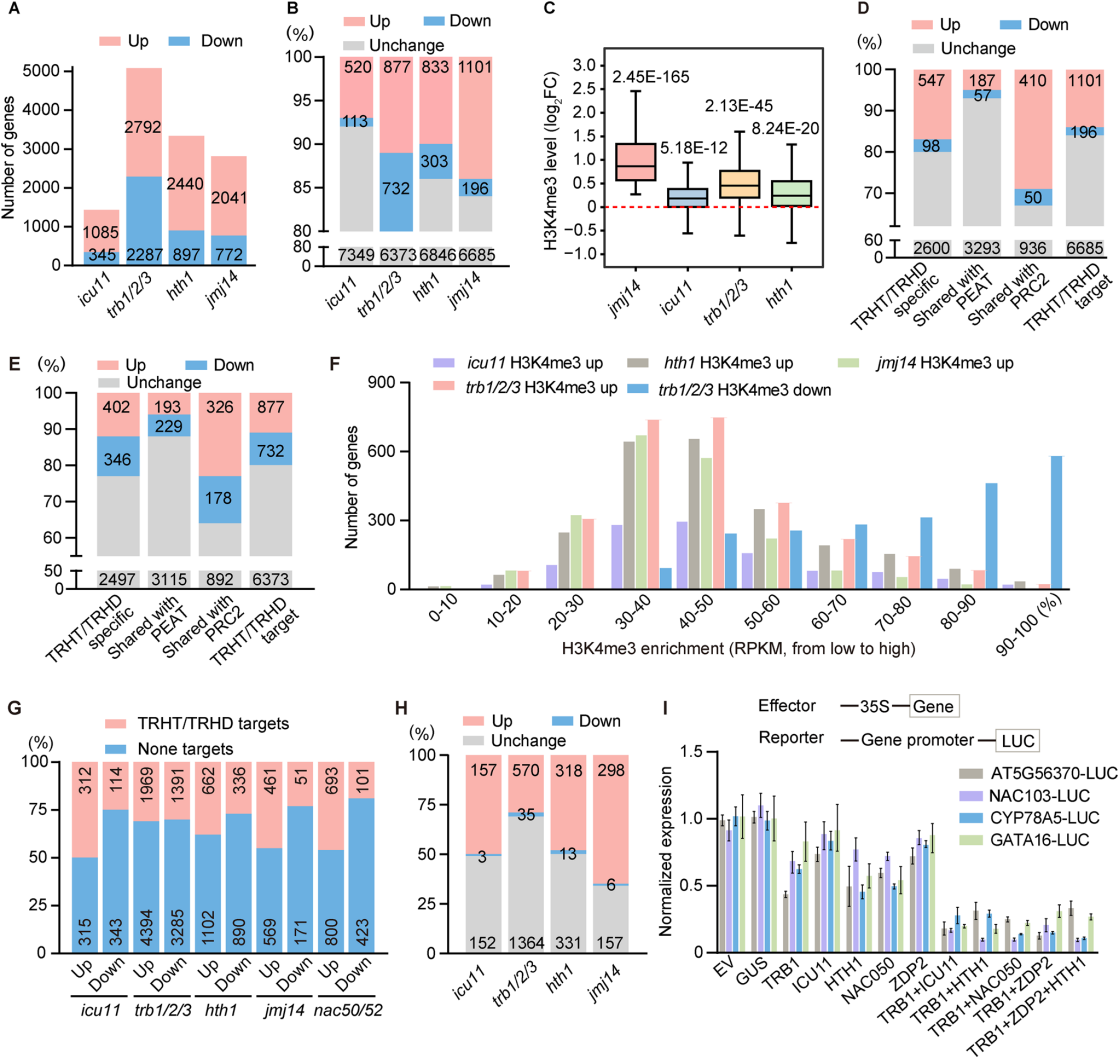
The TRHT/TRHD complexes mediate transcription repression via histone demethylation‐dependent and ‐independent mechanisms. A) Number of genes with up‐ and down‐regulated H3K4me3 levels in the indicated mutants compared to the wild type. B) Percentage of TRHT/TRHD target genes (7982) with increased, reduced, and unchanged H3K4me3 levels in *icu11*, *trb1/2/3*, *hth1*, and *jmj14* mutants. Values represent the number of genes. C) Box plots showing the change of H3K4me3 levels in *jmj14*, *icu11*, *trb1/2/3*, and *hth1* mutants compared to the wild type. Analysis was performed on 1101 genes with increased H3K4me3 levels in the *jmj14* mutant. *P* values were determined by the Mann‐Whitney U test (paired) for non‐normally distributed data. D,E) Number of TRHT/TRHD target genes with up‐ and down‐regulated H3K4me3 levels in (D) *jmj14* and E) *trb1/2/3* mutants compared to the wild type. TRHT/TRHD target genes (7982) were classified into three subgroups for analysis: TRHT/TRHD‐specific genes (3245), genes shared by PEAT (3537), and genes shared by PRC2 (1396). TRHT/TRHD targets represent shared target genes of TRHT and TRHD (7982), which are defined as those co‐enriched by ZDP2, ICU11, TRB1, and HTH1. F) Graph illustrating the distribution of genes with H3K4me3 changes in the indicated mutants relative to the wild type across gene deciles. Total Arabidopsis genes (n = 32,548) were divided into deciles sorted by ascending H3K4me3 levels. G) Ratios of TRHT/TRHD targets and non‐targets among up‐ and down‐regulated DEGs in each mutant. TRHT/TRHD targets (n = 7982) are defined as those co‐bound by ZDP2, ICU11, TRB1, and HTH1. H) Number of up‐regulated TRHT/TRHD target DEGs exhibiting up‐regulated, down‐regulated, and unchanged H3K4me3 levels in *icu11*, *trb1/2/3*, *hth1*, and *jmj14* mutants. I) Determination of the effect of TRHT/TRHD components on transcription by the *LUC* reporter assay in Arabidopsis protoplasts. Shown are a schematic representation of constructs used in the assay (top) and statistical results of relative LUC activity (bottom). Normalized expression levels are presented as the ratio of LUC to REN. Values are means ± SD from three biological replicates. *P* values were determined by the two‐tailed Student's t‐test. EV, empty vector.

JMJ14 has been identified as a demethylase of H3K4me3, a marker associated with transcriptional activation.^[^ ^40^, ^54^ ^]^ We hypothesized that the transcriptional repression function of the TRHT/TRHD complexes is at least partially mediated by JMJ14‐dependent H3K4me3 demethylation. To test this hypothesis, we assessed whether the levels of H3K4me3 were altered in mutants of the TRHT/TRHD complexes compared to the wild‐type control. Western blot analysis indicated that the genome‐wide H3K4me3 levels were not significantly affected in the TRHT/TRHD mutants (Figure S9A, Supporting Information). Additionally, we performed H3K4me3 ChIP‐seq to evaluate whether the H3K4me3 levels at specific TRHT/TRHD target genes were influenced in the TRHT/TRHD mutants, including *icu11*, *trb1/2/3*, *hth1*, and *jmj14*. The ChIP‐seq analysis identified numerous genes with either increased or decreased levels of H3K4me3 (FDR < 0.05, FC > 1.2 or < 0.8) in the TRHT/TRHD mutants when compared to the wild type. Notably, in all these mutants, the number of upregulated genes consistently exceeds that of downregulated genes (**Figure** **4**A; Dataset S5, Supporting Information), suggesting that the TRHT/TRHD complexes can mediate H3K4me3 demethylation at specific genes.

To investigate whether the TRHT/TRHD complexes directly contribute to H3K4me3 demethylation, we analyzed the ratios of genes with increased versus decreased levels of H3K4me3 among the 7982 TRHT/TRHD target genes. The results showed that the proportion of genes with elevated H3K4me3 levels was greater than that of genes with reduced levels in all TRHT/TRHD mutants (Figure 4B). In particular, compared to the other TRHT/TRHD mutants, the *jmj14* mutant exhibited the highest number of TRHT/TRHD target genes with increased H3K4me3 levels (Figure 4B). For the TRHT/TRHD target genes, the H3K4me3 levels were significantly increased in the *icu11*, *trb1/2/3*, and *hth1* mutants relative to the wild type, although the increase was less pronounced than in the *jmj14* mutant (Figure 4C). These results confirm that the TRHT/TRHD complexes can contribute to JMJ14‐dependent H3K4me3 demethylation at their target genes.

We plotted correlation diagrams for gene expression changes versus H3K4me3 alterations across TRHT/TRHD target genes in the *icu11*, *hth1*, *trb1/2/3*, and *jmj14* mutants. In the *icu11*, *hth1*, and *jmj14* mutants, genes with increased expression markedly outnumbered those with decreased expression (Figure 4A), and this increased expression was positively correlated with elevated H3K4me3 levels (Figure S9B, Supporting Information), supporting the notion that TRHT/TRHD complexes mediate transcriptional repression through H3K4me3 demethylation. In the *trb1/2/3* mutant, the number of genes with increased expression was comparable to that of genes with decreased expression (Figure 4A). Genes with either increased or decreased expression showed a positive correlation with H3K4me3 alterations (Figure S9B, Supporting Information). Given that TRB1/2/3 are shared subunits of both the TRHT/TRHD and PEAT complexes, we hypothesize that TRB1/2/3 function as subunits of TRHT/TRHD to mediate H3K4me3 demethylation and transcriptional repression, while acting as subunits of PEAT to mediate transcriptional activation and thereby indirectly enhance H3K4me3.

To explore how TRHT/TRHD complexes select a subset of their target genes for H3K4me3 demethylation, we evaluated the JMJ14‐dependent H3K4me3 demethylation across three subclasses of TRHT/TRHD target genes, categorized based on their overlap with PEAT and PRC2 target genes. Compared to the ratio of the genes with JMJ14‐dependent H3K4me3 demethylation in the TRHT/TRHD‐specific target genes (547/3245, 16.86%), the ratio of these genes was markedly reduced (187/3537, 5.29%) in the TRHT/TRHD‐ and PEAT‐shared target genes, and increased (410/1396, 29.37%) in the TRHT/TRHD‐ and PRC2‐shared target genes (Figure 4D). Similarly, the ratios of TRB1/2/3‐, ICU11‐, and HTH1‐dependent H3K4me3 demethylation genes were also substantially reduced in the TRHT/TRHD‐ and PEAT‐shared target genes and increased in the TRHT/TRHD‐ and PRC2‐shared target genes, as compared to the ratios of those in the TRHT/TRHD‐specific target genes (Figure 4E; Figure S9C,D, Supporting Information). These results suggest that the H3K4me3 demethylation mediated by the TRHT/TRHD complexes is enhanced at PRC2 target genes, while it is diminished at PEAT target genes.

Given that TRB1/2/3 are also components of PRC2 complex responsible for mediating H3K27me3,^[^ ^46^ ^]^ we investigated whether TRB1/2/3‐mediated H3K4me3 demethylation is accompanied by H3K27me3 deposition. Using previously published H3K27me3 ChIP‐seq data in the *trb1/2/3* mutant,^[^ ^46^ ^]^ we found that the ratio of genes with TRB1/2/3‐mediated H3K27me3 deposition was higher at TRHT/TRHD‐ and PRC2‐shared target genes compared to other TRHT/TRHD target genes (Figure S9E, Supporting Information). For other TRHT/TRHD target genes, the genes with TRB1/2/3‐mediated H3K27me3 deposition were also more abundant than those with increased H3K27me3 (Figure S9E, Supporting Information). These results suggest that TRHT/TRHD‐mediated H3K4me3 demethylation is coupled with H3K27me3 deposition.

Furthermore, we quantified the number of genes with JMJ14‐, TRB1/2/3‐, ICU11‐, and HTH1‐dependent H3K4me3 demethylation across gene deciles sorted by H3K4me3 levels. The results indicated that JMJ14 primarily mediates H3K4me3 demethylation at genes with low‐to‐medium levels of H3K4me3 (Figure 4F). This is consistent with the observation that H3K4me3 demethylation is enhanced when the TRHT/TRHD target genes overlap with the PRC2 target genes, which exhibit low H3K4me3 levels. Unlike the *jmj14*, *icu11*, and *hth1* mutants, in which the number of genes with increased H3K4me3 levels greatly exceeded that of genes with reduced H3K4me3 levels (Figure 4A), in the *trb1/2/3* mutant, the number of genes with up‐regulated H3K4me3 levels was only slightly higher than that of genes with down‐regulated H3K4me3 levels (Figure 4A). This suggests that TRB1/2/3 can also contribute to H3K4me3 deposition. We therefore quantified the genes with down‐regulated H3K4me3 levels in the *trb1/2/3* mutant across gene deciles sorted by H3K4me3 levels. The results indicated that these genes were enriched in the deciles with high levels of H3K4me3 (Figure S9F, Supporting Information). Given that TRB1/2/3 are also components of the PEAT complex, which mediates histone acetylation, H2A deubiquitination, and transcriptional activation at genes with high levels of H3K4me3,^[^ ^24^ ^]^ the TRB1/2/3‐dependent deposition of H3K4me3 is likely attributed to the role of TRB1/2/3 in the PEAT complex. These results suggest that the TRHT/TRHD complexes are coordinated with the PEAT and PRC2 complexes to regulate H3K4me3 throughout the entire genome.

### 2.6 H3K4me3 Demethylation‐Dependent and ‐Independent Roles of TRHT/TRHD

To investigate whether JMJ14‐dependent H3K4me3 demethylation contributes to TRHT/TRHD‐mediated transcriptional repression, we performed a combinatorial analysis of RNA‐seq data and H3K4me3 ChIP‐seq data. This analysis indicated that the proportion of direct target genes was consistently higher among up‐regulated DEGs than among down‐regulated DEGs (Figure 4G). Additionally, the enrichment levels of TRHT/TRHD components were significantly higher in up‐regulated DEGs than in down‐regulated DEGs (Figure S10A, Supporting Information). These results support the notion that the TRHT/TRHD complexes are involved in transcriptional repression at their target genes. Among the TRHT/TRHD target genes showing increased expression in the TRHT/TRHD mutants, 28.95% (570/1969) to 64.64% (298/461) exhibited elevated levels of H3K4me3, strongly supporting the hypothesis that JMJ14‐dependent H3K4me3 demethylation plays a critical role in transcriptional repression. Nevertheless, a substantial subset of these target genes with increased expression did not display corresponding increases in H3K4me3 levels in the TRHT/TRHD mutants (Figure 4H). This finding suggests that the TRHT/TRHD complexes can also suppress transcription through mechanisms independent of histone demethylation.

To determine whether the TRHT/TRHD complexes can directly mediate transcriptional repression, we generated a *LUC* reporter driven by the promoters of representative TRHT/TRHD target genes in Arabidopsis mesophyll protoplasts and then assessed whether overexpression of TRHT/TRHD components affects reporter gene expression. Our analysis showed that overexpression of individual TRHD or TRHT components either weakly repressed *LUC* reporter expression or had no effect, whereas co‐overexpression of TRB1 with one or two additional TRHT/TRHD components significantly enhanced their repressive effect (Figure 4I). These results confirm that the TRHT/TRHD complexes can mediate transcriptional repression. To explore whether TRHT/TRHD‐mediated transcriptional repression is linked to JMJ14‐dependent H3K4me3 demethylation, we selected TRHT/TRHD target genes representing two categories for *LUC* reporter assays: those exhibiting H3K4me3 demethylation‐dependent transcriptional repression (e.g., *AT5G56370* and *NAC103*) and those showing H3K4me3 demethylation‐independent repression (e.g., *GATA16* and *CYP78A5*) (Figure S10B, Supporting Information). We found that the TRHT/TRHD complexes consistently exerted transcriptional repression across all these target gene promoters (Figure 4I). These results suggest that the TRHT/TRHD components can cooperate to mediate transcriptional repression in an H3K4me3 demethylation‐independent manner.

### 2.7 NAC052, TRB1, and HTH1 Cooperatively Bind to DNA

**Figure 5:**
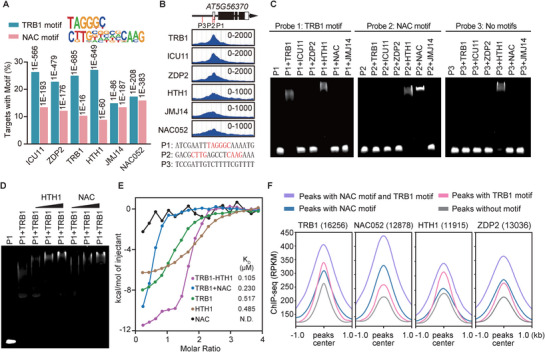
Combinatorial analysis of the DNA‐binding abilities of TRB1, HTH1, and NAC050/052 in vitro and in vivo. A) Bar plots showing the enrichment of putative TRB1 motif and NAC motif in the indicated ChIP‐seq peaks formed by TRHT/TRHD components. The statistical results are sourced from HOMER. *P* values were determined by the hypergeometric test. NAC motif, motif enriched by NAC052 in ChIP‐seq. TRB1 motif, motif enriched by TRB1 in ChIP‐seq. B) Genome browser view of ChIP‐seq signals of TRHT/TRHD components at a representative gene. The scale of reads per kilobase of exon per million reads mapped (RPKM) is indicated for each panel. P1, P2, and P3 are three probes used for EMSA. The TRB1 motif in P1 and the NAC motif in P2 are labeled in red. C) The binding of TRHT/TRHD components to DNA as determined by EMSA. Probe 1 and 2 contain the TRB1 motif and NAC motif, respectively, while Probe 3 does not contain the TRB1 motif or the NAC motif. NAC, NAC050‐N. D) Determination of the binding of TRB1 to DNA in the presence or absence of HTH1 and NAC. The P1 probe contains the TRB1 motif. NAC, NAC050‐N. E) Determination of TRB1‐DNA binding affinity by ITC in the presence or absence of NAC050‐N and HTH1. NAC indicates the N‐terminal region of NAC050 (NAC050‐N). (F) Meta plots showing the distribution of ChIP‐seq signals for TRB1 (16256), NAC052 (12878), HTH1 (11915), and ZDP2 (13036) across their respective peaks, categorized by the presence of the NAC motif and/or the TRB1 motif.

In the TRHT/TRHD complexes, NAC050/052 and TRB1/2/3 have been identified as transcriptional repressors that bind to DNA in a sequence‐dependent manner.^[^ ^38^, ^44^, ^46^ ^]^ To assess the contribution of NAC050/052 and TRB1/2/3 to the association of the TRHT/TRHD complexes with chromatin, we performed HOMER annotations for the ChIP‐seq peaks of TRHT/TRHD components. HOMER‐annotated TRB1‐binding motifs exhibited sequence identity to the established *telobox* motif, which is known to be bound by TRB1.^[^ ^46^ ^]^ Our analysis revealed that the peaks of these components were enriched for both the TRB1 motif and the NAC motif (**Figure** **5**A). Notably, TRB1, ICU11, ZDP2, and HTH1 showed greater enrichment for the TRB1 motif compared to the NAC motif, whereas JMJ14 and NAC052 exhibited equivalent enrichment for both motifs (Figure 5A). This observation is consistent with previous findings that the binding of NAC050/052 to its motif is crucial for the association of JMJ14 and NAC050/052 with chromatin.^[^ ^38^, ^44^ ^]^ Furthermore, we found that the proportion of TRHT/TRHD‐binding genomic regions containing both the TRB1 motif and the NAC motif was significantly higher than expected by chance (Figure S11A, Supporting Information). These results suggest that TRB1/2/3 and NAC050/052 have a synergistic effect on the association of the TRHT/TRHD complexes with chromatin.

To further investigate how the TRHT/TRHD components cooperate to bind target genomic loci, we selected a TRHT/TRHD target locus that contains both the TRB1 motif and the NAC motif for Electrophoretic Mobility Shift Assays (EMSA) (Figure 5B). The EMSA results indicated that TRB1/2/3 and NAC050 specifically bound to the TRB1 motif (probe 1) and the NAC motif (probe 2), respectively, while HTH1/2/3 exhibited sequence‐independent binding to DNA (Figure S11B, Supporting Information). Additionally, other TRHT/TRHD components, including ICU11, ZDP2, and JMJ14, did not show any DNA‐binding ability (Figure 5C). Therefore, TRB1/2/3 may collaborate with NAC050/052 and HTH1/2/3 within the TRHD and TRHT complexes, respectively, to bind their target genomic loci.

To explore how TRB1/2/3 cooperate with NAC050/052 and HTH1/2/3 in DNA binding, we co‐incubated TRB1 with either NAC050‐N or HTH1 for EMSA. We found that while TRB1 alone could bind to the DNA containing the TRB1 motif, the addition of the TRB1‐interacting proteins NAC050‐N or HTH1 resulted in the formation of a larger complex and enhanced the DNA‐binding capacity of TRB1 on this DNA (Figure 5D,E). Similarly, the addition of TRB1 to NAC050‐N promoted the formation of a larger complex on the DNA containing the NAC motif (Figure S11C, Supporting Information). These results demonstrate that TRB1 can cooperate with NAC050 and HTH1 to bind DNA in vitro.

To validate this cooperation in vivo, we classified the ChIP‐seq peaks of TRB1, NAC052, ZDP2, and HTH1 into four groups based on the presence or absence of the NAC motif or the TRB1 motif. Subsequently, we assessed their enrichment levels across these groups (Figure 5F). This analysis revealed that peaks containing both the NAC and TRB1 motifs exhibited the highest enrichment, whereas the enrichment was substantially reduced in the TRB1 and NAC050 peaks lacking either of these motifs and further decreased in those lacking both motifs. Consistent with the composition of the TRHT complex, which includes TRB1 but not NAC050/052, the TRHT‐specific subunit HTH1 showed significantly greater enrichment at peaks harboring the TRB1 motif than those containing the NAC motif (Figure 5F). Conversely, in line with the TRHD complex containing both NAC050/052 and TRB1, the TRHD‐specific subunit ZDP2 exhibited comparable enrichment levels between peaks with the TRB1 motif and those with the NAC motif (Figure 5F). As a shared subunit of both complexes, TRB1 displayed similar enrichment across peaks containing either motif (Figure 5F). Notably, despite NAC052 being a TRHD‐specific subunit, its enrichment was significantly higher at peaks containing the NAC motif than those with the TRB1 motif (Figure 5F), suggesting that the sequence‐dependent DNA binding capacity of NAC052 plays a primary role in mediating its chromatin association.

The NAC family transcription factors typically bind to DNA as dimers.^[^ ^55^, ^56^ ^]^ In this study, we used the AlphaFold3 server to predict the structure of a DNA‐protein complex formed by four proteins (two copies of NAC050/052 and one copy each of HTH1 and TRB1) bound to a DNA segment containing both TRB1 and NAC motifs.^[^ ^57^ ^]^ The predicted model revealed that TRB1 recognizes and anchors into the TRB1 motif region via its N‐terminal domain, while two NAC050/52 molecules bind to the DNA in a symmetric conformation, with the NAC domain of one NAC050/052 directly interacting with the NAC motif (Figure S12A, Supporting Information). To identify key DNA‐protein binding sites, we focused on amino acid sites located within 5 Å of the two types of DNA motifs. In the TRB1 motif region, the N‐terminal Myb domain of TRB1 interacts with bases G20’, C21’, and C22’ in the reverse strand of the major groove through residues K5, K50, and N55, while NAC050/052 engages with T24’ and A25’ in the reverse strand of the minor groove via R133, and A7 in the forward strand of the major groove via A137 (Figure S12B, Supporting Information), which is consistent with in vitro binding assays showing that NAC050/052 enhances the DNA‐binding ability of TRB1 (Figure 5D,E). In the NAC motif region, one NAC050/052 molecule binds to multiple bases in the major groove, including A6’, G7’, T19, G20, and A21. This interaction is mediated by residues M134, R136, T146, and R150 within its NAC domain (Figure S12B, Supporting Information). Additionally, the GH1 domain of TRB1 contacts T25 in the minor groove via residue T176 (Figure S12B, Supporting Information). These structural predictions support the notion that NAC050/052 and TRB1 can form a complex to simutatenously bind DNA in vivo.

### 2.8 The Myb and GH1 Domains are Involved in the Association of TRB1 with Chromatin

**Figure 6:**
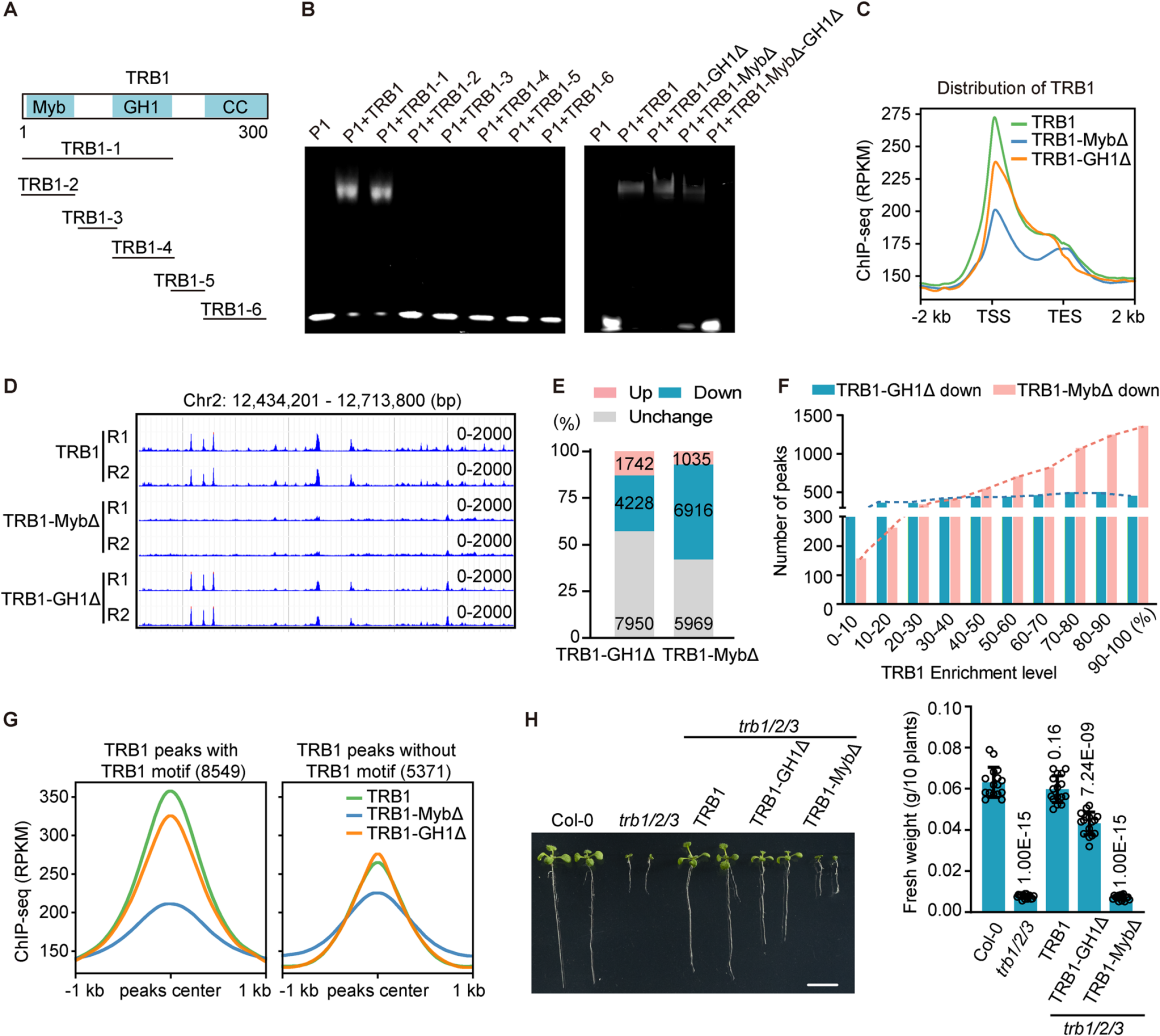
Effect of the Myb and GH1 domains on the binding of TRB1 to DNA. A) Schematic representation of truncated TRB1 proteins used in EMSA. B) The binding of truncated TRB1 proteins to DNA as determined by EMSA. TRB1‐MybΔ, Myb‐deleted TRB1; TRB1‐GH1Δ, GH1‐deleted TRB1; TRB1‐ MybΔ‐GH1Δ, Myb‐ and GH1‐deleted TRB1. The P1 probe containing a TRB1 motif was used in EMSA. (C) Metaplots showing the ChIP‐seq signal of wild‐type TRB1, TRB1‐MybΔ, and TRB1‐GH1Δ over protein‐coding genes. TSS, transcription start site; TES, transcription end site. The ChIP‐seq data are from two independent biological replicates. D) Genome browser view of ChIP‐seq signals of wild‐type TRB1, TRB1‐MybΔ, and TRB1‐GH1Δ. Two biological replicates (R1 and R2) are shown. The scale of RPKM is labeled. E) Percentage of TRB1 peaks with up‐regulated, down‐regulated, and unchanged enrichment of TRB1‐MybΔ and TRB1‐GH1Δ compared to TRB1 enrichment. F) Number of TRB1 peaks with reduced TRB1‐MybΔ and TRB1‐GH1Δ enrichment in the deciles of TRB1 peaks with ascending levels of TRB1 enrichment. G) Meta plots showing the distribution of wild‐type TRB1, TRB1‐MybΔ, and TRB1‐GH1Δ ChIP‐seq signals over the TRB1 peaks with (left) and without (right) the TRB1‐binding motif. H) Determination of the effect of the Myb and GH1 deletion on plant development by complementation testing. The morphological phenotype (left) and the statistical results of the plant fresh weight (right) are shown in wild type (Col‐0), *trb1/2/3*, and transgenic plants expressing TRB1‐Flag, TRB1‐MybΔ‐Flag, or TRB1‐GH1Δ‐Flag in the *trb1/2/3* mutant background. Scale bar, 1 cm. Mean values and standard deviation (SD) are calculated from at least 16 plants. *P* values were determined by the two‐tailed Student's t‐test.

The TRB1/2/3 proteins contain a conserved N‐terminal Myb domain, followed by a putative GH1 domain and a C‐terminal coiled‐coil domain (**Figure** **6**A; Figure S13, Supporting Information). The Myb domain and the GH1 domain in other proteins have been shown to mediate sequence‐dependent DNA binding and nucleosome dyad binding, respectively.^[^ ^58^, ^59^, ^60^, ^61^ ^]^ Consequently, we purified a series of truncated TRB1 variants (TRB1‐1 ∼ TRB1‐6) and assessed their DNA‐binding capabilities using EMSA (Figure 6A). The EMSA results demonstrated that the truncated TRB1 fragment TRB1‐1, which contains both the Myb and GH1 domains, was capable of binding to the TRB1 motif‐containing probe P1 (Figure 6B). In contrast, the other truncated variants did not bind to the probe (Figure 6A,B). Furthermore, we generated Myb and/or GH1‐deleted forms of TRB1 and evaluated their DNA‐binding abilities via EMSA. The results indicated that the GH1‐deleted form (TRB1‐GH1Δ) retained its DNA‐binding ability, whereas the Myb‐deleted form (TRB1‐MybΔ) showed a partial reduction in DNA binding (Figure 6B). The TRB1 variant lacking both the Myb and GH1 domain (TRB1‐MybΔ‐GH1Δ) completely lost its ability to bind DNA (Figure 6B). These in vitro experiments suggest a cooperative role of the Myb and GH1 domains in DNA binding.

To investigate the contributions of the Myb and GH1 domains to the association of TRB1 with chromatin in vivo, we performed ChIP‐seq using transgenic plants expressing TRB1‐MybΔ‐Flag, TRB1‐GH1Δ‐Flag, and wild‐type TRB1‐Flag. While the wild‐type TRB1 exhibited a pronounced peak around the TSS, the TRB1‐MybΔ and to a lesser extent TRB1‐GH1Δ peaks were markedly lower than the wild‐type TRB1 peak (Figure 6C,D). We identified numerous TRB1 peaks where enrichment was affected by the deletion of the Myb and GH1 domains, with a majority of the affected peaks showing decreased enrichment (Figure 6E; Dataset S6, Supporting Information). The TRB1 peaks affected by the Myb deletion showed relatively high enrichment levels, whereas those affected by the GH1 deletion exhibited a range of enrichment levels (Figure 6F). Additionally, the Myb deletion exhibited a substantial effect on the enrichment of the TRB1 peaks containing the TRB1 motif while only showing a weak effect on the enrichment of the TRB1 peaks lacking this motif (Figure 6G). These results indicate that the Myb domain is primarily responsible for the sequence‐dependent binding of TRB1 to highly enriched regions.

Moreover, we conducted a complementation test to determine whether the Myb and GH1 deletions affect the biological function of TRB1 in Arabidopsis. While the wild‐type *TRB1* transgene fully complemented the developmental defects observed in the *trb1/2/3* mutant, the *TRB1‐MybΔ* transgene failed to complement these defects, whereas the *TRB1‐GH1Δ* transgene partially restored normal development (Figure 6H). These results support the idea that the Myb domain and to a lesser extent the GH1 domain mediate the association of TRB1 with chromatin, thereby contributing to plant development in Arabidopsis.

## 3 Discussion

**Figure 7:**
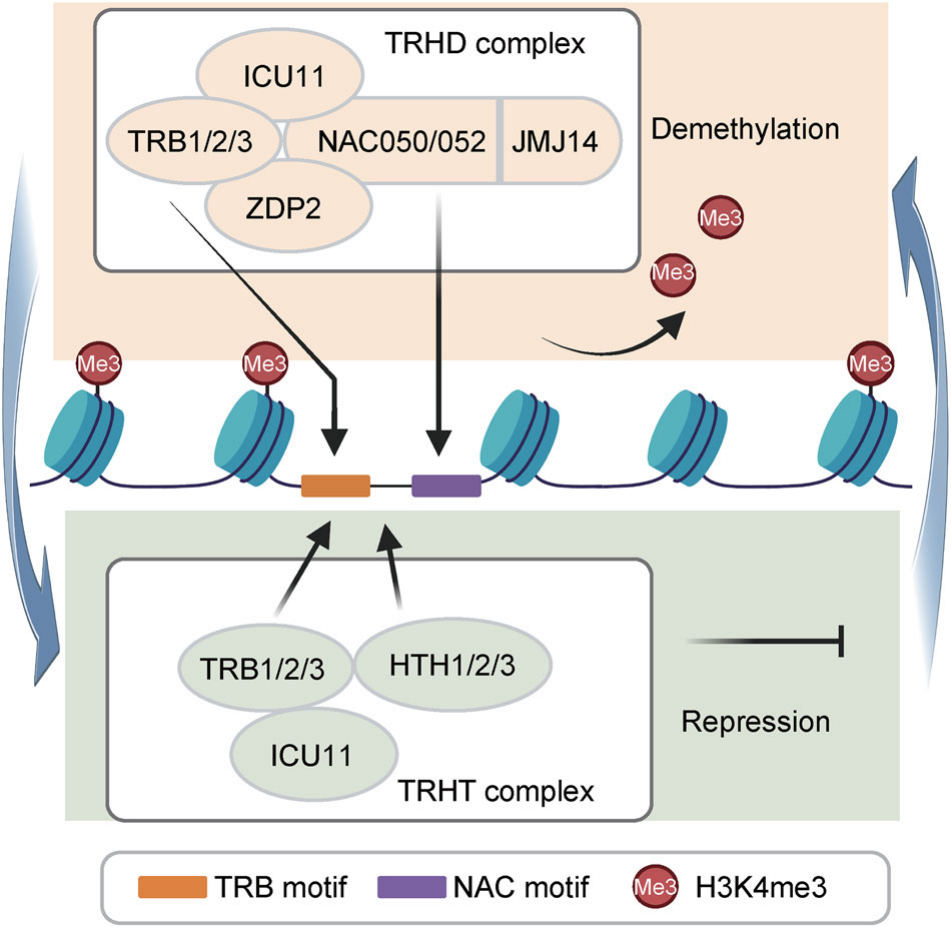
Working model illustrating roles of TRHD and TRHT complexes in histone demethylation and transcriptional repression. The TRHD complex, which consists of ICU11, TRB1/2/3, ZDP2, NAC050/052, and JMJ14, facilitates JMJ14‐mediated H3K4me3 demethylation. Within the TRHD complex, the sequence‐dependent DNA‐binding transcriptional repressors TRB1/2/3 and NAC050/052 bind to the TRB motif and the NAC motif, respectively, thereby facilitating the association of the TRHD complex with chromatin. The TRHT complex, composed of TRB1/2/3, HTH1/2/3, and ICU11, directly represses transcription. In the TRHT complex, the sequence‐dependent DNA‐binding transcriptional repressor TRB1/2/3 and the sequence‐independent DNA‐binding proteins HTH1/2/3 cooperate to mediate the association of the TRHT complex with chromatin. The TRHT and TRHD complexes form a positive feedback loop, which synergistically contributes to H3K4me3 demethylation and transcriptional repression.

This study identifies two closely related chromatin‐associated complexes, TRHT and TRHD, and demonstrates that they can cooperate to mediate transcriptional repression at common target genes. The identification of TRHT and TRHD as distinct complexes relies on the finding of the mutual exclusivity between the TRHT‐specific subunits HTH1/2/3 and the TRHD‐specific subunits JMJ14, NAC050/052, and ZDP2. However, the complexes contain two types of shared components, TRB1/2/3 and ICU11, indicating a close relationship between them. The close relationship between the two complexes is further supported by the finding that they target common target genomic loci and co‐regulate histone demethylation and transcriptional repression (**Figure** **7**). Given that TRB1/2/3 possess a sequence‐dependent DNA‐binding ability,^[^ ^60^, ^61^ ^]^ the DNA‐binding ability of TRB1/2/3 likely plays a major role in mediating the association of both the TRHT and TRHD complexes with chromatin. Our findings demonstrate that the Myb domain is critical for the association of TRB1 with chromatin in a sequence‐dependent manner. In addition, the TRHT components HTH1/2/3 function as sequence‐independent DNA‐binding proteins, while the TRHD components NAC050/052 act as sequence‐dependent transcriptional repressors.^[^ ^38^, ^44^ ^]^ ICU11, which lacks direct DNA‐binding ability, has been shown to interact with the PRC2 complex, which primarily mediates H3K27 trimethylation in intragenic regions. The relatively high enrichment level of ICU11 in intragenic regions is likely due to its association with the PRC2 complex.

Considering that the TRHT and TRHD complexes primarily target common genomic loci, we predict that the DNA‐binding ability of TRB1/2/3 plays a major role in targeting the TRHT and TRHD complexes to specific genomic loci, while HTH1/2/3 and NAC050/052 provide an additional layer for targeting selection. It is more likely that HTH1/2/3 and NAC050/052 may either strengthen the association of their corresponding complexes with their common target genomic loci or contribute to the association of the complexes with their respective target genomic loci. The high degree of overlap between the TRHT target genes and the TRHD target genes indicates that TRB1/2/3, as their shared components, play a major role in mediating the association of the TRHT and TRHD complexes with their common target genomic loci, while the TRHT‐specific components HTH1/2/3 and the TRHD‐specific components NAC050/052 provide an additional layer for their respective targeting selection (Figure 7). Both the TRHT and TRHD components can mediate transcriptional repression through both H3K4me3 demethylation‐dependent and ‐independent mechanisms, suggesting these complexes may form a positive feedback loop to mediate H3K4me3 demethylation and transcriptional repression at their common target genes (Figure 7). Consistent with this model, a recent study showed that artificial recruitment of HTH2/TRBIP1 to specific loci effectively removes H3K4me3 and promotes DNA methylation,^[^ ^39^ ^]^ supporting the idea that TRHT can cooperate with TRHD to facilitate H3K4me3 demethylation. However, it remains unclear whether TRHT and TRHD bind to their common target genomic loci simultaneously within the same nucleus or independently across different nuclei. In the future, single‐cell multi‐omics approaches could provide deeper insights into the spatiotemporal dynamics of TRHT and TRHD cooperation.

Consistent with the previous finding that JMJ14‐mediated H3K4me3 demethylation occurs at genes with low to medium levels of H3K4me3,^[^ ^39^ ^]^ our study indicates that all the tested components of the TRHT and TRHD complexes are capable of mediating H3K4me3 demethylation at genes with low‐to‐medium levels of H3K4me3, thereby confirming that these complexes can mediate demethylation in a JMJ14‐dependent manner. This raises a question regarding how JMJ14 mediates H3K4me3 demethylation at low‐to‐medium levels of H3K4me3. In the human H3K4me3 demethylase KDM5A, a PHD domain specifically recognizes unmodified H3 and thereby stimulates the histone demethylase activity of KDM5A,^[^ ^62^ ^]^ revealing a molecular mechanism underlying the positive feedback of H3K4me3 demethylation. We suggest that JMJ14 or other components of the TRHD complex may recognize the nucleosome with unmodified H3K4me3, thereby facilitating its histone demethylase activity on neighboring H3K4me3 nucleosomes. Alternatively, JMJ14 may preferentially mediate H3K4me3 demethylation on the nucleosome with one copy of H3K4me3 rather than on those with two copies of H3K4me3. Our study supports the idea that JMJ14 facilitates the propagation of H3K4me3 demethylation on chromatin with pre‐existing low levels of H3K4me3 through a positive‐feedback loop.

We find that the JMJ14‐dependent H3K4me3 demethylation is inhibited at the TRHD target genes shared by the PEAT complex and is promoted at the TRHD target genes shared by the PRC2 complex. Considering that the PEAT complex is responsible for histone acetylation and H2A deubiquitination,^[^ ^25^ ^]^ we predict that the H3K4me3 demethylase activity of JMJ14 is inhibited by either high levels of histone acetylation or low levels of H2A ubiquitination mediated by the PEAT complex. Additionally, JMJ14‐dependent H3K4me3 demethylation is likely enhanced by PRC2‐mediated H3K27me3. In humans, the binding of the H3K9me3 demethylase KDM4C to H3K4me3 through its tandem Tudor domains stimulates its H3K9me3 demethylation activity.^[^ ^63^, ^64^ ^]^ Given that the JMJ14‐mediated H3K4me3 demethylation is enhanced at H3K27me3‐enriched chromatin regions, we predict that H3K27me3 may enhance the association of JMJ14 with chromatin or its histone demethylase activity and thereby exclude H3K4me3 from the H3K27me3‐enriched chromatin regions. Similarly, although the TRHT complex does not contain JMJ14, it seems to create a chromatin environmental condition that benefits the association of the TRHD complex with chromatin or the H3K4me3 demethylase activity of JMJ14, thereby contributing to JMJ14‐mediated H3K4me3 demethylation.

Our study reveals that TRB1/2/3, as shared subunits of the PEAT, TRHT/TRHD, and PRC2 complexes, can coordinate the regulation of multiple histone modifications. The PEAT complex mediates histone acetylation and H2A deubiquitination at target genes with high levels of H3K4me3, whereas the PRC2 complex mediates deposition of H3K27me3 at target genes with low levels of H3K4me3. Different from the PEAT and PRC2 target genes, the TRHT/TRHD complexes mediate H3K4me3 demethylation at target genes with low‐to‐medium levels of H3K4me3. In Arabidopsis, H3K4me3 and H3K27me3 are mutually exclusive at the whole‐genome level.^[^ ^65^, ^66^, ^67^ ^]^ Our study indicates that the removal of methylation from H3K4me3 by the TRHT/TRHD complexes is coupled with the deposition of H3K27me3 mediated by the PRC2 complex, which synergistically contributes to the formation of the repressive chromatin state. In contrast, we find that the H3K4me3 demethylation mediated by the TRHT/TRHD complexes is suppressed at the PEAT target genes, suggesting that the H3K4me3 demethylation role of the TRHT/TRHD complexes is inhibited either by high levels of histone acetylation or by low levels of H2A ubiquitination. Therefore, the presence of the TRHT/TRHD complexes significantly increases the regulatory complexity of the chromatin state, facilitating the formation of a hierarchy of chromatin states at the whole‐genome level.

Although JMJ14 acts as a component of the TRHD complex, it specifically regulates flowering time, which differs from the other TRHD components, TRB1/2/3, ICU11, and ZDP2, which are involved in various developmental processes. This suggests that the TRHD complex can also repress gene transcription in an H3K4me3 demethylation‐independent manner. This notion is supported by the combinatorial analysis of RNA‐seq and H3K4me3 ChIP‐seq data at the TRHD target genes. Further studies are needed to determine how the TRHD target genes undergo transcriptional repression without affecting H3K4me3. Previous studies suggest that NAC050/052, as transcriptional repressors, can directly repress gene transcription,^[^ ^44^, ^68^ ^]^ providing a plausible explanation for the H3K4me3 demethylation‐independent role of TRHD in transcriptional repression. Similarly, TRB1/2/3 have also been found to function as transcriptional repressors,^[^ ^50^, ^69^ ^]^ although the molecular mechanisms underlying the role of TRB1/2/3 in transcriptional repression need to be elucidated. Within the TRB1/2/3 proteins, the conserved GH1 domain is highly similar to the GH1 domain in the linker histone H1.^[^ ^59^ ^]^ The H1 is involved in compacting chromatin into a higher‐order structure, which prevents gene transcription.^[^ ^70^, ^71^, ^72^ ^]^ In Arabidopsis, H1 was shown to enhance chromatin condensation and H3K27me3 deposition at PRC2 target genes, while preventing H3K27me3 deposition at telomeres and large pericentromeric interstitial telomeric repeat domains by restricting DNA accessibility to TRB proteins.^[^ ^73^ ^]^ Given that TRB proteins are core components of TRHT/TRHD complexes, further studies should investigate how H1 and TRB proteins coordinate at TRHT/TRHD target genes to regulate chromatin status and transcription.

## 4 Experimental Section

### Plant Materials and Constructs

All Arabidopsis plants investigated in this study are in the Col‐0 ecotype. The Arabidopsis seedlings were cultivated on Murashige and Skoog (MS) medium plates in the growth chamber with a long‐day photoperiod (16 h light, 23 °C; 8 h dark, 22 °C). The *trb1* (Salk_025147), *trb2‐2* (GABI_103E02), and *jmj14* (Salk 135712C) mutants used in this study were T‐DNA insertion mutants obtained from the Arabidopsis Biological Resource Center. The *nac050/052* double mutant was generated in the published work.^[^ ^44^ ^]^ Through CRISPR‐Cas9‐mediated genome editing, *icu11*, *zdp2*, *hth1*, *hth2/trbip1*, and *hth3* single mutants and a *trb1/2/3* triple mutant were obtained in the wild‐type background. The primers used in genotyping are listed in Dataset S7 (Supporting Information).

To obtain the transgenic plants, the full‐length genomic sequence of *TRB1*, *ICU11*, *ZDP2*, *HTH1*, *HTH2*, and *HTH3*, including the native promoter, was cloned into a modified pCAMBIA1305 vector with a 30‐terminal FLAG tag. To generate TRB1‐MybΔ and TRB1‐GH1Δ, the truncated genomic sequences TRB1‐MybΔ (residues 61–300 aa) and TRB1‐GH1Δ (residues 1–113 and 186–300 aa) with their native promoters were inserted into the pCAMBIA1305 vector with a 3 × Flag at their 3’ terminus. The constructs were introduced into the *Agrobacterium*, which was used to transform Arabidopsis plants by the flower‐dipping method. The constructs were verified by sequencing, and the primers used in the cloning are listed in Dataset S7 (Supporting Information).

### Affinity Purification and Mass Spectrometry Analysis

Affinity purification was conducted with 8 g of plant materials, including 12‐day‐old transgenic seedlings and flower tissues. These materials were ground into powder and suspended in 20–25 ml lysis buffer (50 mm Tris‐HCl [pH 7.5], 150 mm NaCl, 5 mm MgCl_2_, 10% glycerol, 0.1% NP‐40, 1 mm dithiothreitol [DTT], 1 mm phenylmethylsulfonyl fluoride [PMSF], and 0.1% Roche protease inhibitor cocktail tablet). The resuspended material was then rotated at 4 °C for 30 min. Subsequently, the solution was filtered through a Miracloth (475855; Merck Millipore) and subjected to two rounds of centrifugation at 12 000 rpm for 15 min. The supernatant was then combined with anti‐FLAG M2 agarose (A2220; Sigma) and incubated at 4 °C overnight. The protein‐bound beads were washed four times with lysis buffer and then eluted for 1 h with lysis buffer containing 3×FLAG peptide (F4799; Sigma). The eluted proteins were separated on a 10% SDS‐PAGE gel and stained with the ProteoSilver silver stain kit (PROT‐SIL2; Sigma). The mass spectrometry analysis was conducted as previously described.^[^ ^25^, ^74^ ^]^ The heatmap displaying the AP‐MS results was generated by using GraphPad Prism (version 8). The protein‐protein interaction diagram, as determined by AP‐MS, was constructed by using Cytoscape (v3.6.1) software.

### Nucleocytoplasmic Separation

Sucrose density gradient centrifugation was used to separate the nuclei and cytoplasm of Arabidopsis plants. One gram of 12‐day transgenic material was ground into powder with liquid nitrogen. The powder was then transferred into lysis buffer (20 mm Tris‐HCl [pH 7.5], 20 mm KCl, 2 mm EDTA, 2.5 mm MgCl_2_, 25% [v/v] glycerol, 250 mm sucrose, and 5 mm DTT) and rotated at 4 °C for 30 min. The solution was then filtered through Miracloth and centrifuged at 4000 rpm for 10 min. The supernatant was transferred to a new tube and centrifuged at 12 000 rpm for 15 min, and the supernatant was the cytoplasm. The pellet was resuspended with NRBT buffer (20 mm Tris‐HCl [pH 7.5], 2.5 mm MgCl_2_, 25% [w/v] glycerol, and 0.2% [v/v] Triton X‐100) and then centrifuged at 4000 rpm for 10 min. This step was repeated three times. The pellet was resuspended with NEB2 (20 mm Tris‐HCl [pH 7.5], 10 mm MgCl_2_, 250 mm sucrose, 0.5% [v/v] Triton X‐100, and 5 mm β‐mercaptoethanol) and then gently transferred to NEB3 (20 mm Tris‐HCl [pH 7.5], 10 mm MgCl_2_, 1.7 m sucrose, 0.5% [v/v] Triton X‐100, and 5 mm β‐mercaptoethanol) at 12 000 rpm for 45 min, and the pellet was the nuclei. The cytoplasm and nuclei obtained from the above steps were used for western blotting to detect the subcellular localization of the TRB1, ICU11, ZDP2, HTH1, JMJ14, and NAC052 proteins. UGPase (Abcam, ab154817) and H3 (Abcam, ab1791) represent markers in the cytoplasm and nucleus, respectively.

### Yeast Two‐Hybrid Assay

For yeast two‐hybrid assays, the full‐length coding sequences of *TRB1*, *TRB2*, *TRB3*, *ICU11*, *ZDP2*, *HTH1*, *HTH2*, *HTH3*, *JMJ14*, and *NAC050* and *NAC052* were cloned into pGADT7 and pGBKT7 vectors by using a One‐Step Cloning Kit (C112‐01; Vazyme). The primers utilized in the cloning process are listed in Dataset S7 (Supporting Information). The recombinant vectors were transferred to yeast strains AH109 or Y187 and screened on a synthetic dropout medium lacking leucine (SD‐L) or tryptophan (SD‐W). The positive colonies were mixed and mated overnight at room temperature. The mixture was then spread on a synthetic dropout medium lacking leucine and tryptophan (SD‐LW) to screen positive colonies. Positive colonies were subsequently resuspended in sterilized double‐distilled water (ddH_2_O) and then spotted on the synthetic dropout medium lacking leucine, tryptophan, and histidine (SD‐LWH) but containing 0 or 3 mm 3‐amino‐1,2,4‐triazole (3‐AT).

### Protein Purification and Pull‐Down Assay

The full‐length of *ICU11*and *ZDP2* with GST tag were cloned into the pGEX6P‐1 vector, the full‐length of *TRB1*, *TRB2*, *TRB3*, *HTH1*, *HTH2*, and *HTH3* with His tag were cloned into the pSMT3 vector, and the full‐length of *ICU11*, *ZDP2*, *HTH1*, *TRBIP1*, *HTH3*, and *NAC050* with MBP tag were cloned into the pET‐30a vector. The primers used are listed in Dataset S7 (Supporting Information). The constructs were transformed into the *Escherichia coli* strain BL21. Positive colonies were cultured in LB liquid medium to an OD600 of 0.6‐0.8. After centrifugating at 4000 rpm for 20 min, the collected bacteria were resuspended with GST‐tag lysis buffer (20 mm Tris–HCl [pH 7.5], 150 mm NaCl, 1 mm DTT, and 1 mm PMSF), His‐tag lysis buffer (20 mm Tris–HCl [pH 7.5], 500 mm NaCl, 20 mm imidazole, 1 mm DTT, and 1 mm PMSF), or MBP‐tag lysis buffer (20 mm Tris–HCl [pH 7.5], 200 mm NaCl, 1 mm EDTA, 1 mm DTT, and 1 mm PMSF). GST beads (GE Healthcare, 17‐0756‐01), His beads (Millipore, 70666‐4), or MBP beads (Smart‐lifesciences, SM035001) were added to the supernatant after sonication and centrifugation. After 1 h of incubation, the beads were washed five times and then eluted with GST‐tag lysis buffer containing 20 mm reduced glutathione, His‐tag lysis buffer containing 250 mm imidazole, or MBP‐tag elution buffer (20 mm Tris–HCl [pH 7.4], 10 mm maltose, 1 mm EDTA) for 1 h and centrifuged to collect the supernatant. The supernatant was the purified protein.

The full length of JMJ14 with FLAG tag was cloned into the pAT424 vector with a 30‐terminal FLAG tag. The construct was transformed into the yeast strain YPH499 for protein purification. The yeast pellets were ground in liquid nitrogen and resuspended in yeast lysis buffer (50 mm Tris‐HCl [pH 7.4], 150 mm NaCl, 1 mm EDTA, 10% glycerol, 0.05% NP‐40, 1 mm DTT, 1 mm PMSF, and 0.1% Roche protease inhibitor cocktail tablet). After centrifugation at 12 000 rpm for 15 min, the supernatants were incubated with anti‐Flag M2 agarose (Sigma, A2220). The resins were washed five times with yeast lysis buffer. Flag‐tagged proteins were eluted with 3 × Flag peptides. Eluted proteins were purified proteins.

For the pull‐down assay, the proteins were mixed in GST‐tag lysis buffer at 4 °C for 1 h. After removing 10% of the mixture as an input, the remaining mixture was incubated with 10% GST or MBP beads at 4 °C for 1 h. After incubating, the beads were washed five times with the GST‐tag lysis buffer and eluted with GST‐tag or MBP‐tag elution buffer. The elution was analyzed by immunoblotting using GST antibody (Abmart, M20007L), MBP antibody (Abclonal, AE016), or His antibody (ORIGENE, TA150088).

### Gel Filtration

Gel filtration was performed as previously described.^[^ ^75^ ^]^ In brief, TRB1‐His, NAC050‐MBP, ZDP2‐MBP, ICU11‐GST, HTH1‐His, and JMJ14‐Flag were mixed and incubated at 4 °C overnight. The mixture was then centrifuged at 12 000 rpm for 1 min. The supernatant was filtered through a 0.22‐µm membrane and then loaded onto a Superose 6 column (10/300GL; GE Healthcare, 17‐5172‐01). Eluates were collected, separated by SDS‐PAGE gel, and detected by immunoblotting using antibodies against GST (Abmart, M20007L), FLAG (Abclonal, AE005), MBP (Abclonal, AE016), or HIS (ORIGENE, TA150088).

### RNA‐Seq and Data Analysis

0.1 g of 12‐day seedlings were extracted with TRIzol reagent (15596018; Ambion) to generate total RNA and then sent to Novogene for library preparation and deep sequencing by the paired‐end scheme (PE150) on the Illumina NovaSeq 6000 instrument. After removal of adapters and low‐quality reads, the clean reads were mapped to the Arabidopsis genome (TAIR 10) with HISAT2 (v2.1.0).^[^ ^76^ ^]^ The DEGs were identified with a threshold (HDR < 0.05, |log_2_FC| > 0.5) with the R (v4.3.0) package edgeR (v3.42.4).^[^ ^77^ ^]^ The results in this study were from three independent biological replicates.

The GO analysis was performed through the online website DAVID (https://david.ncifcrf.gov/tools.jsp). The heatmap of DEGs was drawn by using the R package ggplots (v3.1.3.1). The bubble charts were drawn using the R package ggplot2 (v3.5.0).

### EMSA

The synthesis‐produced Cy5‐labeled oligonucleotides were annealed to form a double‐stranded DNA. The recombinant protein was purified from *E. coli*. In the EMSA, 20 ng of the labeled DNA was incubated with 1 µg of the fusion protein in binding buffer (10 mm Tris‐HCl [pH 7.5], 50 mm NaCl, 1 mm EDTA, 5% glycerol, and 1 mm DTT) at 25 °C for 30 min, and was then run on 6% nondenaturing PAGE gel at 80 V for 1.5 h to separate.

### ChIP‐Seq and Data Analysis

The ChIP‐seq was performed according to the previous study.^[^ ^35^ ^]^ In brief, 4 g of 12‐day‐old seedling material was cross‐linked with 1% formaldehyde under vacuum. The samples were ground into powder and suspended with buffer I (0.4 m sucrose, 10 mm Tris‐HCl [pH 7.5], 1 mm PMSF, 1 mm EDTA, 1 mm DTT, and 1% Roche protease inhibitor cocktail tablet). After filtering with two‐layer Miracloth and centrifugation at 4000 rpm for 20 min, the pellets were washed with buffer II (0.25 m sucrose, 10 mm Tris‐HCl [pH 7.5], 10 mm MgCl_2_, 1% TritonX‐100, 0.1 mm PMSF, 1 mm DTT, and 1% Roche protease inhibitor cocktail tablet) until the pellet was gray. Then, the pellet was resuspended in buffer III (1.7 m sucrose, 10 mm Tris‐HCl [pH 7.5], 10 mm MgCl_2_, 0.15% Triton X‐100, 0.1 mm PMSF, 1 mm DTT, and 1% Roche protease inhibitor cocktail tablet). After centrifugation at 12 000 rpm for 1 h, the pellet was resuspended with sonication buffer (1 mm EDTA [pH 8.0], 10 mm Tris‐HCl [pH 7.5], and 0.25% SDS) and sonicated by the Bioruptor sonicator. After sonication, samples were centrifuged at 12 000 rpm for 15 min, and then the supernatant was diluted with dilution buffer (16.7 mm Tris‐HCl [pH 7.5], 167 mm NaCl, 1.1% Triton X‐100, and 1.2 mm EDTA). The diluted supernatant was incubated with H3K4me3 antibody (Abcam, ab8580) coupled with Dynabeads Protein A (10002D; Thermo Fisher) or with FLAG antibody (F1804; Sigma) coupled with Dynabeads Protein G (10004D; Thermo Fisher) at 4 °C overnight. The beads were washed with low‐salt buffer (150 mm NaCl, 0.1% SDS, 1% Triton X‐100, 2 mm EDTA, and 20 mm Tris–HCl [pH 7.5]), high‐salt buffer (500 mm NaCl, 0.1% SDS, 1% Triton X‐100, 2 mm EDTA, and 20 mm Tris–HCl [pH 7.5]), LiCl buffer (0.25 m LiCl, 1% NP‐40, 1% SDS, 1 mm EDTA, and 10 mm Tris‐HCl [pH 7.5]), and TE buffer (1 mm EDTA and 10 mm Tris‐HCl [pH 7.5]). The protein‐DNA binding complex was eluted with elution buffer (1% SDS and 0.1 m NaHCO_3_). The eluted DNA was purified by extraction with phenol/chloroform/isoamyl alcohol reagent.

Purified DNA was sent to Novogene for library preparation and deep sequencing (Illumina NovaSeq 6000, PE150). For data analysis, the clean data were mapped to the Arabidopsis genome by Bowtie2 (v2.3.4) allowing one mismatch.^[^ ^78^ ^]^ Probable PCR duplicates were removed by using Picard Tools (v2.23.0) with Mark Duplicates. The differentially enriched peaks of H3K4me3 between the mutants and the wild‐type control were identified by SICER2 (v1.0.2) with a threshold (FDR < 0.05, FC > 1.2 or < 0.8).^[^ ^79^ ^]^ The enriched peaks of TRHT/TRHD components were identified by MACS2 (v2.2.7.1), and input reads were used as a negative control.^[^ ^80^ ^]^ The specific enriched peaks were identified by setting a threshold FC greater than 1.5. The read counts were normalized to reads per kilobase per million mapped reads by the number of clean reads mapped to the genome in each library. Motif analysis was done using the findMotifsGenome.pl and scanMotifGenomeWide.pl programs with default parameters in Homer (v4.11.1).^[^ ^81^ ^]^ The ChIP‐seq results in this study were from two independent biological replicates. The correlation heatmap and boxplots were drawn by the R package ggplot2 (v3.5.0). The overlaps of occupied genes were drawn by the R package UpSetR (v1.4.0), ggvenn (v0.1.10), and ggVennDiagram (v1.5.2). The profile plot and heatmap of ChIP‐seq were drawn by DeepTools (v3.5.1).

### Dual‐Luciferase Reporter Assay

The luciferase reporter assay was performed as described with minor modifications.^[^ ^82^, ^83^ ^]^ In brief, effector constructs were generated by cloning TRHT/TRHD component‐encoding genes into the modified PGBKT7 vector under the control of the 35S promoter. A construct expressing 35S‐driven *GUS* gene was used as a negative control. Reporter constructs were generated by cloning the 1.5‐kb promoter regions of TRHT/TRHD target genes (*NAC103*, *AT5G56370*, *GATA16*, and *CYP78A5*) into the pGreen‐0800‐LUC vector upstream of the luciferase gene. Reporter and effector constructs were co‐transformed into Arabidopsis mesophyll protoplasts. The luciferase activity assay was performed using a dual‐luciferase reporter assay system (Promega, E1910).

### ITC Assay

Purified proteins were concentrated and buffer‐exchanged into ITC buffer (20 mm Tris‐HCl at pH 7.5 and 150 mm NaCl) using Amicon Ultra Centrifugal Filters (Millipore, UFC901096) at 4 °C. Proteins were diluted to 5 µm in the ITC buffer, while DNA was dissolved in the same buffer and adjusted to 100 µm. The ITC binding assay was performed using a MicroCal PEAQ‐ITC instrument (Malvern) at 25 °C following the manufacturer's standard protocol. Data were analyzed using the MicroCal PEAQ‐ITC analysis software. DNA oligonucleotides were synthesized by Tsingke Biotechnology.

### Statistical Analysis

The RNA‐seq data were independently repeated in three biological replicates. ChIP‐seq data were obtained from two biological replicates. Phenotypic data were based on at least 15 samples. Data are presented as mean ± SD. Statistical analyses were performed using GraphPad Prism 8 software. For comparisons between two groups with normally distributed data, Student's t‐test was used. For comparisons among three or more groups, one‐way ANOVA followed by Tukey's test was employed. *P* values were determined by the Mann‐Whitney U test (paired) for non‐normally distributed data. Statistical significance was defined as *P* < 0.05. Detailed statistical methods for each experiment are provided in the corresponding figure legends or methods.

### Accession Numbers

The Arabidopsis sequence data employed in this study are derived from TAIR10. The accession numbers of genes reported in this study are as follows: *TRB1* (AT1G49950), *TRB2* (AT5G67580), *TRB3* (AT3G49850), *ICU11* (AT1G22950), *ZDP2* (AT3G17460), *HTH1* (AT2G17540), *HTH2/TRBIP1* (AT4G35510), *HTH3* (AT5G66000), *JMJ14* (AT4G20400), *NAC050* (AT3G10480), *NAC052* (AT3G10490), *UBP5* (AT2G40930), *ARID2* (AT2G17410), *ARID3* (AT1G20910), *ARID4* (AT1G76510), *EPCR1* (AT4G32620), *EPCR2* (AT5G04670), *HAM1* (AT5G64610), *HAM2* (AT5G09740), *PWWP1* (AT3G03140), *LHP1* (AT5G17690), *MSI1* (AT5G58230), *CLF* (AT2G23380), *FIE1* (AT3G20740), *SWN* (AT4G02020), *EMF2* (AT5G51230).

## Conflict of Interest

The authors declare no conflict of interest.

## Author Contributions

Q.W. and X.J.H. were responsible for the project conception, experimental design, and manuscript preparation. Q.W. carried out the experiments. D.Y.Y. performed the bioinformatic analysis. L.L. and S.C. performed the mass spectrometry analysis.

## Supporting information



Supporting Information

Supporting Information

## Data Availability

Raw ChIP‐seq and RNA‐seq data have been deposited in the Gene Expression Omnibus (GEO) database with the accession code GSE287725.
