# Supplementary material for: Arabidopsis TRB Proteins Form Two Closely Related Complexes to Mediate H3K4me3 Demethylation and Transcriptional Repression
Source: Adv Sci (Weinh). 2025 Oct 5;12(47):e03420. doi: 10.1002/advs.202503420 (PMC12713088; doi:10.1002/advs.202503420)
Supplement: Supplementary file 1 — Supporting Information [file ADVS-12-e03420-s001.pdf]

PEAT | TRHT/TRHD | PRC2

Bait \ Target	EPCR1	EPCR2	PWWP1	ARID2	ARID3	ARID4	HAM1	HAM2	UBP5	TRB1	TRB2	TRB3	HTH1	HTH2	HTH3	NAC050	NAC052	JMJ14	ZDP2	ICU11	CLF	SWN	LHP1	EMF2	MSI1	FIE1
EPCR1	6	0	5	6	5	5	3	3	5	5	3	0	0	0	0	0	0	0	0	0	0	0	0	0	0	0
ARID2	6	0	5	7	5	6	2	2	5	4	1	0	0	0	0	0	0	0	0	0	0	0	0	0	0	0
HAM1	5	2	4	4	2	3	8	0	4	3	0	0	0	0	0	0	0	0	0	0	0	0	0	0	0	0
UBP5	4	0	4	4	3	3	0	0	8	2	0	0	0	0	0	0	0	0	0	0	0	0	0	0	0	0
TRB1	7	6	7	6	5	5	6	0	6	7	2	3	1	0	1	6	6	6	5	5	1	2	3	2	3	0
TRB2	5	4	5	5	3	4	4	0	5	4	7	3	2	4	5	6	6	6	5	6	4	5	5	5	5	5
TRB3	5	3	5	4	4	4	4	2	5	4	4	7	2	4	5	6	6	6	4	6	4	5	4	5	5	6
HTH1	0	0	0	0	0	0	0	0	0	0	1	2	2	0	3	0	0	0	0	2	0	0	0	0	0	0
HTH2	0	0	0	0	0	0	0	0	0	0	2	2	0	5	0	0	0	0	0	2	0	0	0	0	1	0
HTH3	0	0	0	0	0	0	0	0	0	0	3	3	2	0	4	0	0	2	0	3	0	0	0	0	1	0
NAC052	0	0	0	0	0	0	0	0	0	2	0	0	0	0	0	8	8	4	2	3	0	0	0	0	0	0
JMJ14	0	0	0	0	0	0	0	0	0	2	0	0	0	0	0	6	7	10	2	3	0	0	0	0	2	0
ZDP2	0	0	0	0	0	0	0	0	0	5	4	4	0	0	0	5	5	3	6	5	0	0	0	0	0	0
ICU11	0	0	0	0	0	0	0	0	0	5	5	6	4	2	4	5	5	2	5	9	0	3	2	3	0	0
CLF	0	0	0	0	0	0	0	0	0	0	2	3	2	0	0	0	0	0	0	4	6	4	5	5	5	5
SWN	0	0	0	0	0	0	0	0	0	0	3	0	3	0	1	0	0	0	0	3	3	4	2	2	3	3

log_2(Matched queries + 1)

0 1 2 3 4 5

Supplemental Figure 1. Heatmap showing components of the PEAT, TRHT/TRHD, and PRC2 complexes as determined by AP-MS. Color density represents the enrichment of normalized queries identified by AP-MS. Transgenic plants expressing indicated bait proteins in a wild-type background were subjected to AP-MS analysis.

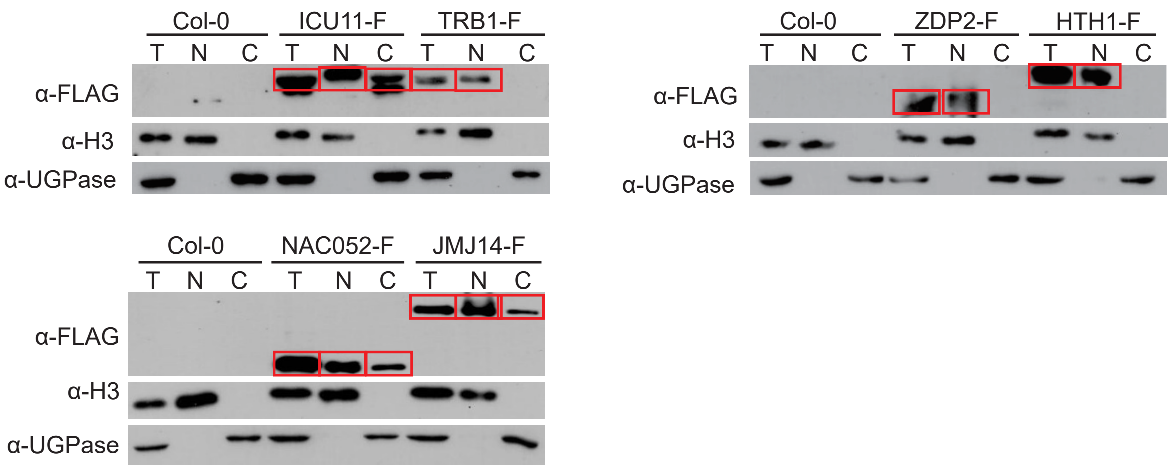

Supplemental Figure 2. Nuclear-cytoplasmic fractionation of TRHT/TRHD components.
Subcellular localization of Arabidopsis TRHT/TRHD components was determined by nuclear-cytoplasmic fractionation followed by western blot analysis. UGPase and H3 serve as cytoplasmic and nuclear markers, respectively. T, total proteins; N, nuclear proteins; C, cytoplasmic proteins. Target protein bands are highlighted with red boxes.

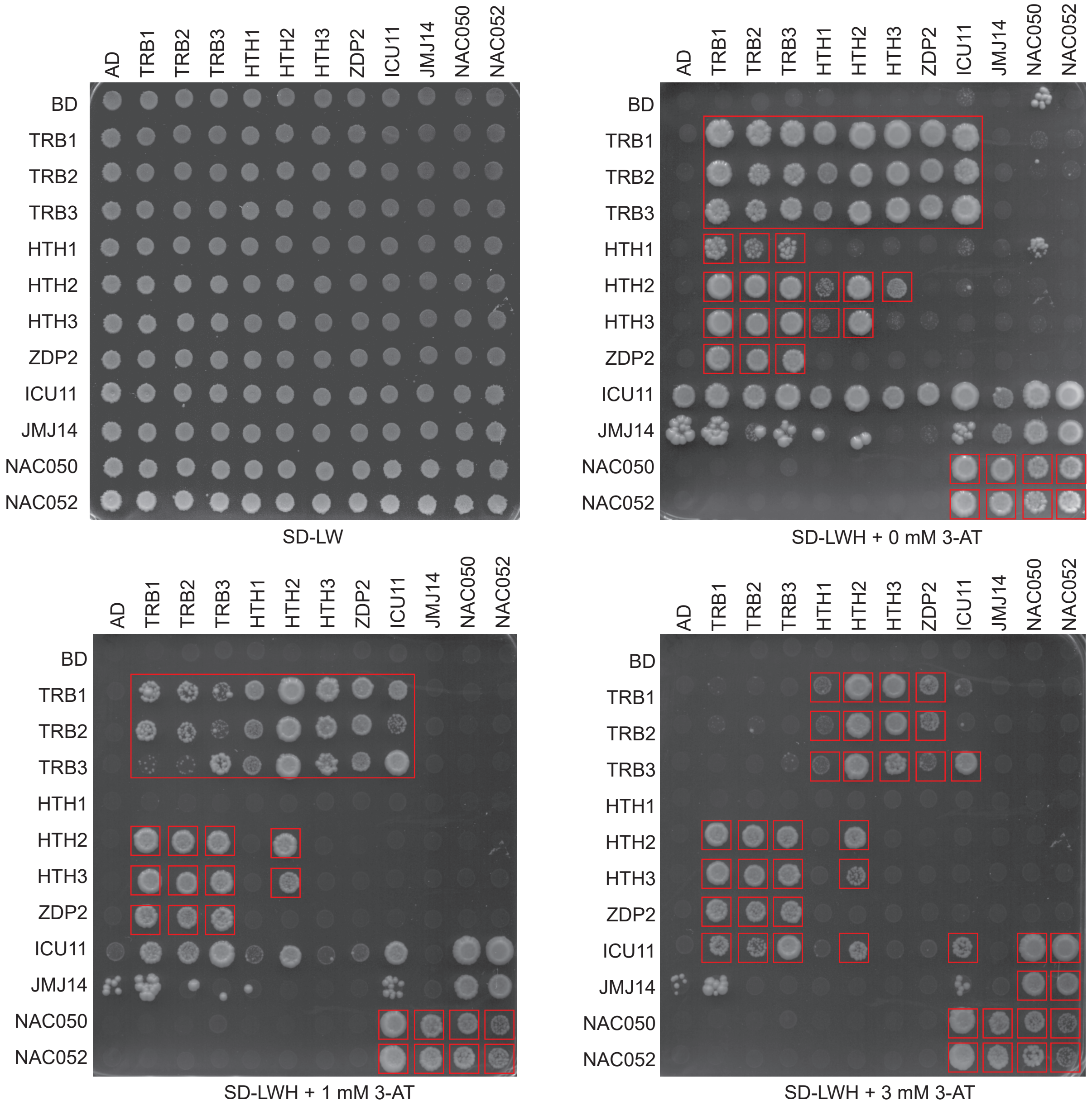

Supplemental Figure 3. Interactions of TRHT/TRHD components determined by yeast two-hybrid assays.
TRHT/TRHD complex components were fused to either the GAL4 DNA-binding domain (GAL4-BD) or the GAL4 transcriptional activation domain (GAL4-AD) for yeast two-hybrid assays. SD-LW, synthetic dropout medium lacking leucine (L) and tryptophan (W); SD-LWH, synthetic dropout medium lacking leucine (L), tryptophan (W), and histidine (H) supplemented with 0, 1, and 3 mM 3-amino-1,2,4-triazole (3-AT).

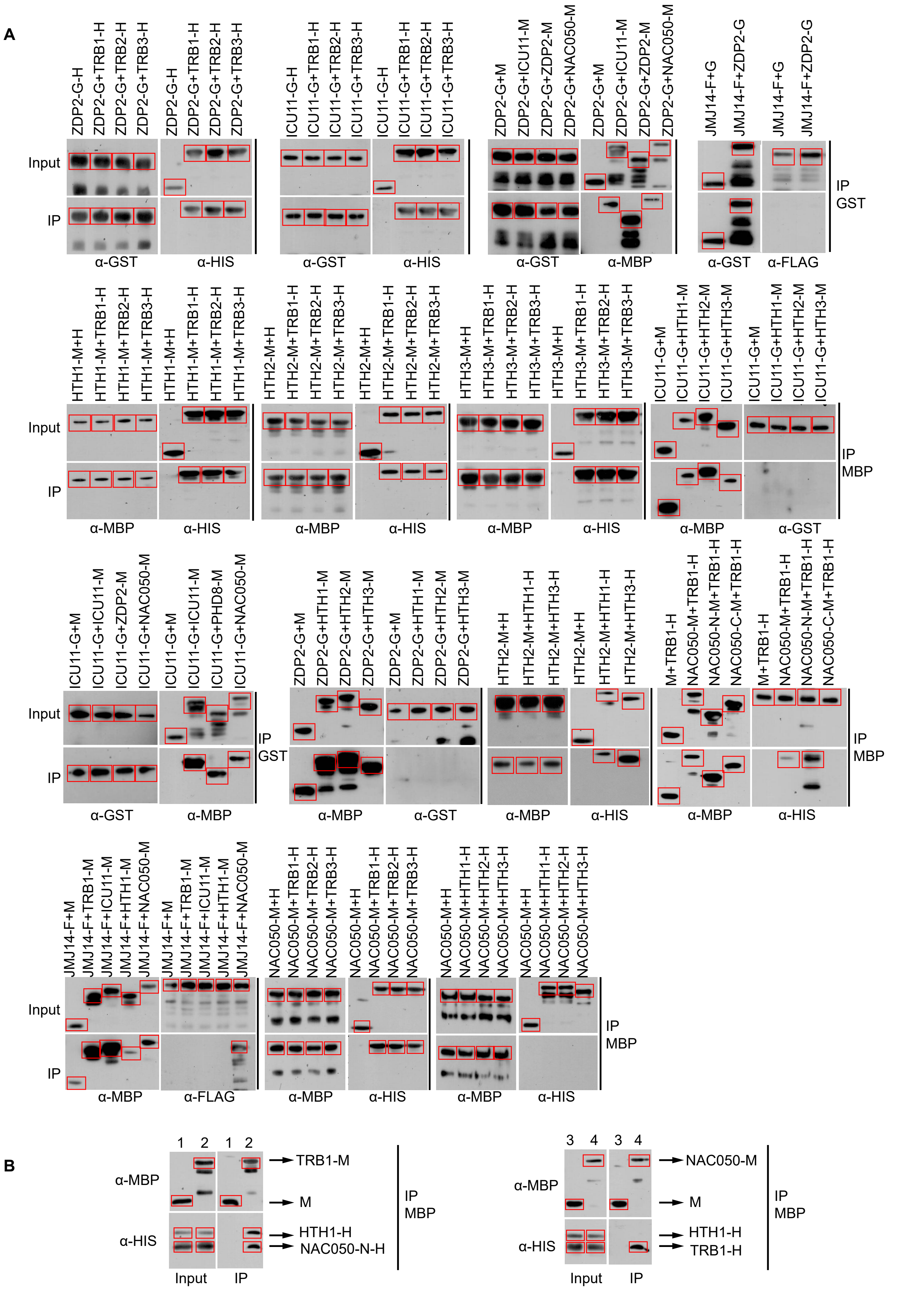

Supplemental Figure 4. Interactions of TRHT/TRHD complex components determined by pull-down assays.
(A) TRHT/TRHD complex components were fused with His, MBP, or GST tags, followed by pull-down assays.
(B) TRB1-dependent interaction between HTH1 and NAC050-N, analyzed by pull-down assay. 1, mixture of MBP, HTH1-His, and NAC050-N-His; 2, mixture of TRB1-MBP, HTH1-His, and NAC050-N-His; 3, mixture of MBP, HTH1-His, and TRB1-His; 4, mixture of NAC050-N-MBP, HTH1-His, and TRB1-His.
For pull-down assays shown in (A) and (B), each pair of tagged proteins was precipitated using antibodies against GST or MBP, and subjected to western blot analysis. Target protein bands were labeled with red boxes. H, His; M, MBP; G, GST.

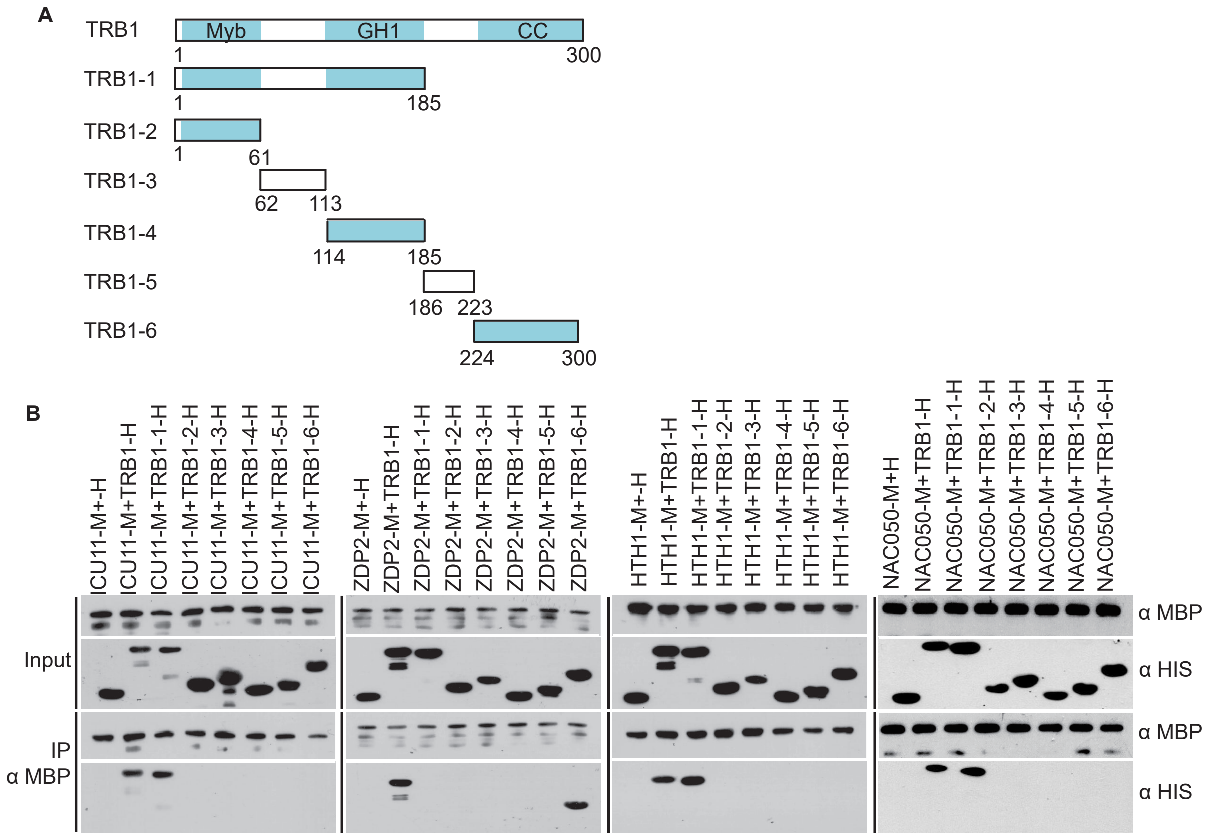

Supplemental Figure 5. Interactions between TRHT/TRHD components and truncated TRB1 variants determined by pull-down assays.
(A) Schematic representation of full-length and truncated versions of TRB1 used in pull-down assays. Start and end sites of each truncated protein are shown.
(B) Interactions of ICU11, ZDP2, HTH1, and NAC050 with truncated versions of TRB1. TRHT/TRHD components ICU11, ZDP2, HTH1, and NAC050 fused to MBP were individually mixed with each truncated version of TRB1, precipitated using anti-MBP antibody-conjugated beads, and analyzed by western blot. H, His; M, MBP.

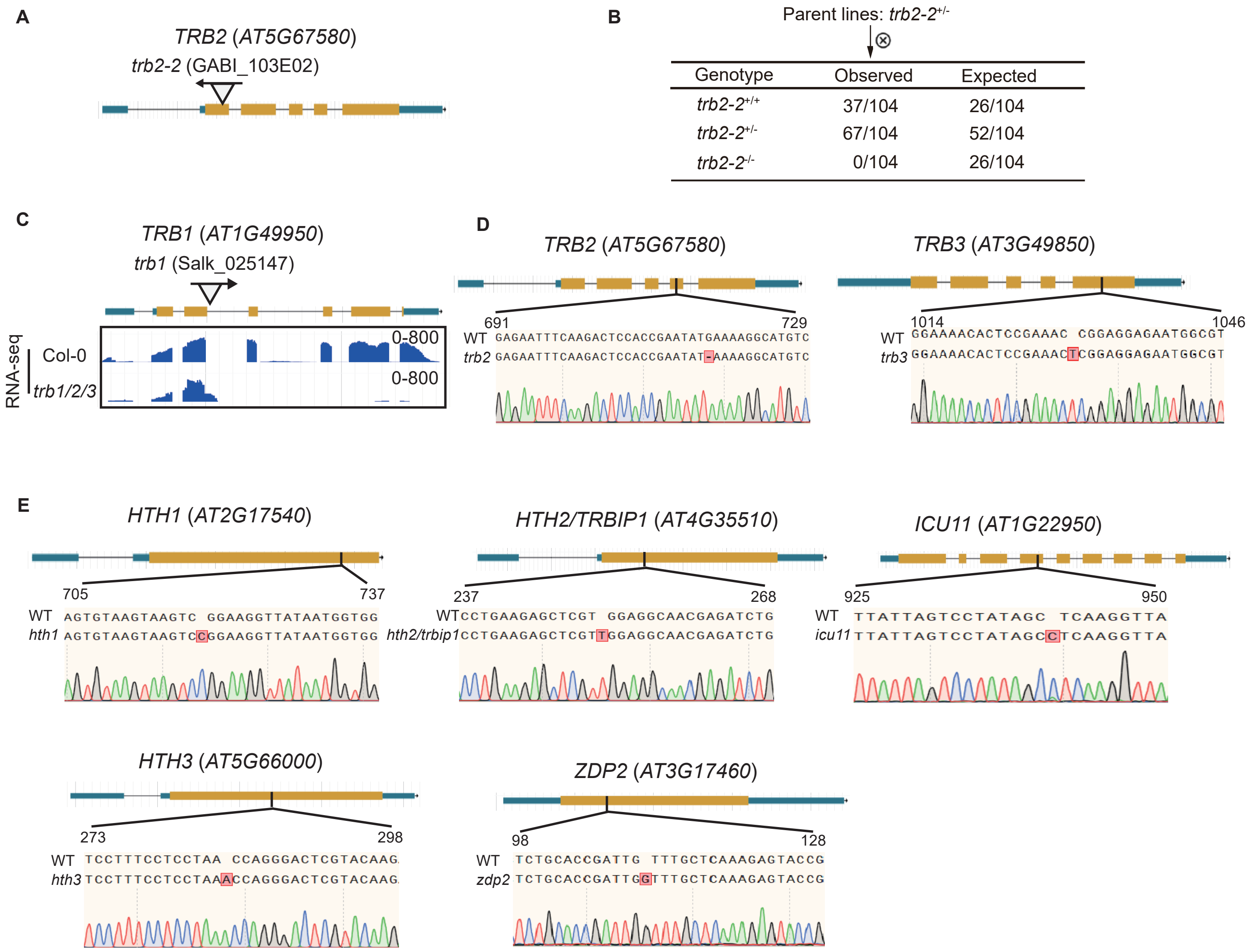

Supplemental Figure 6. Validation of TRHT/TRHD mutants used in this study.
(A) Schematic representation of the *trb2-2* mutant, with the T-DNA insertion site indicated by a triangle.
(B) Genotypes of the progeny from self-bred *trb2-2*$^{+/-}$ plants. Observed and expected numbers of progeny with indicated genotypes are shown.
(C) Genome browser view of *TRB1* RNA-seq signals in Col-0 and the *trb1/2/3* mutant. The RPKM scale is shown.
(D) Schematic representation of *trb2* and *trb3* mutations in the *trb1/2/3* mutant. CRISPR-induced *trb2* and *trb3* mutations are labeled within the guide RNA target sequences. Numbers represent positions relative to the translation start site of coding sequences.
(E) Schematic representation of *hth1*, *hth2*, *hth3*, *icu11*, and *zdp2* mutations. CRISPR-induced mutations are labeled within the guide RNA target sequences. Numbers indicate positions relative to the translation start site of coding sequences.

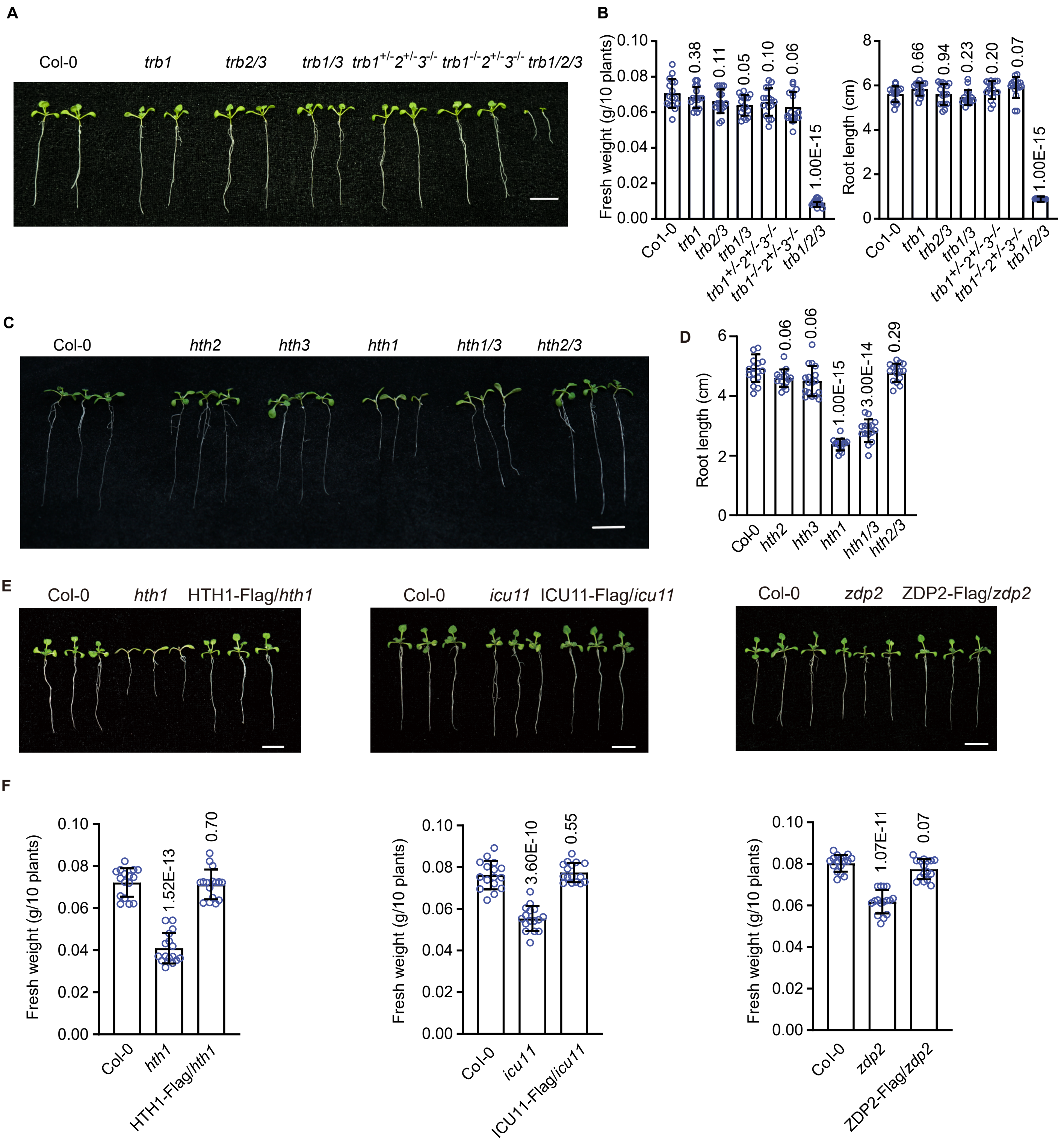

Supplemental Figure 7. Morphological phenotypes of TRHT/TRHD mutants.

(A) Morphological phenotypes of mutants with *trb1*, *trb2*, and *trb3* mutations. The phenotypes of 12-day-old seedlings are shown. Scale bar, 1 cm.

(B) Statistical results of the fresh weight and root length in 12-day-old seedlings. Mean values and standard deviation (SD) are from at least 15 plants. *P* values were determined by the two-tailed Student's t-test.

(C) Morphological phenotypes of mutants with *hth1*, *hth2*, and/or *hth3* mutations. The phenotypes of 12-day-old seedlings are shown. Scale bar, 1 cm.

(D) Statistical results of the root length in 12-day-old seedlings. Mean values and standard deviation (SD) are from at least 15 plants. *P* values were determined by the two-tailed Student's t-test.

(E) Morphological phenotypes of *hth1*, *icu11*, *zdp2*, and their respective complementation lines. The phenotypes of 12-day-old seedlings are shown. Scale bar, 1 cm.

(F) Statistical results of the fresh weight in 12-day-old seedlings. Mean values and standard deviation (SD) are from at least 15 plants. *P* values were determined by the two-tailed Student's t-test.

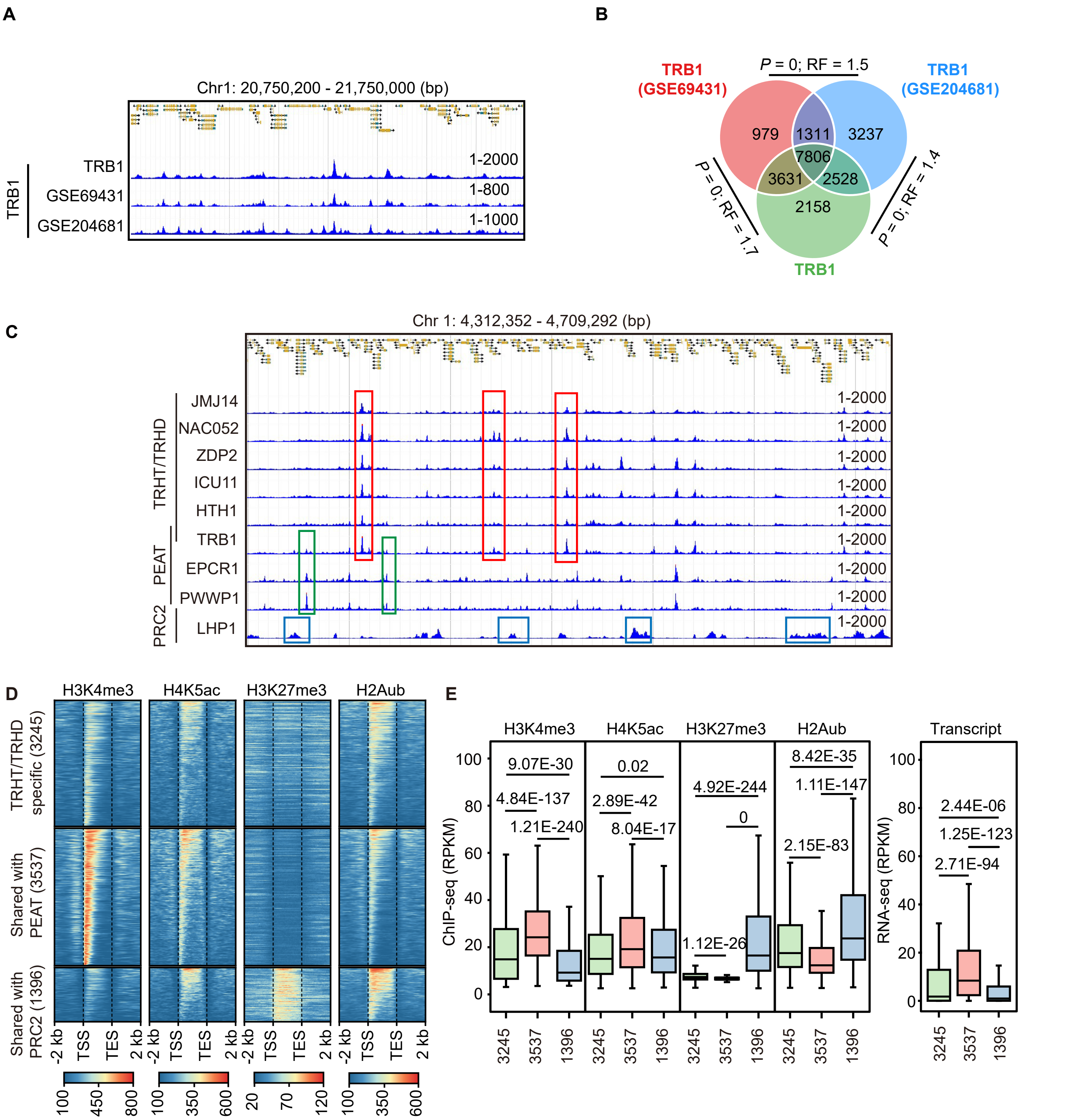

Supplemental Figure 8. Analysis of the ChIP-seq peaks of TRHT/TRHD components.

(A) Comparison of TRB1 ChIP-seq signals from the current study and published data within a representative genomic region. The RPKM scale is shown.

(B) Venn diagram illustrating the overlap of genes bound by TRB1 based on published ChIP-seq datasets (GSE69431 and GSE204681) and the current study.

(C) Genome browser view of ChIP-seq signals for TRHT/TRHD, PEAT, and PRC2 components in a representative genomic region. Red, blue, and green boxes indicate peaks specific to TRHT/TRHD, PRC2, and PEAT complexes, respectively.

(D) Heatmaps displaying ChIP-seq signals of H3K4me3, H4K5ac, H3K27me3, and H2Aub at three subclasses of TRHT/TRHD target genes: TRHT/TRHD-specific target genes (3245), TRHT/TRHD- and PEAT-shared target genes (3537), and TRHT/TRHD- and PRC2-shared target genes (1396). TSS, transcription start site; TES, transcription end site.

(E) Boxplots showing the ChIP-seq enrichment levels of the indicated histone modifications and RNA-seq enrichment level at the three TRHT/TRHD target gene subclasses. *P* values were determined by the two-tailed Mann-Whitney U test (unpaired).

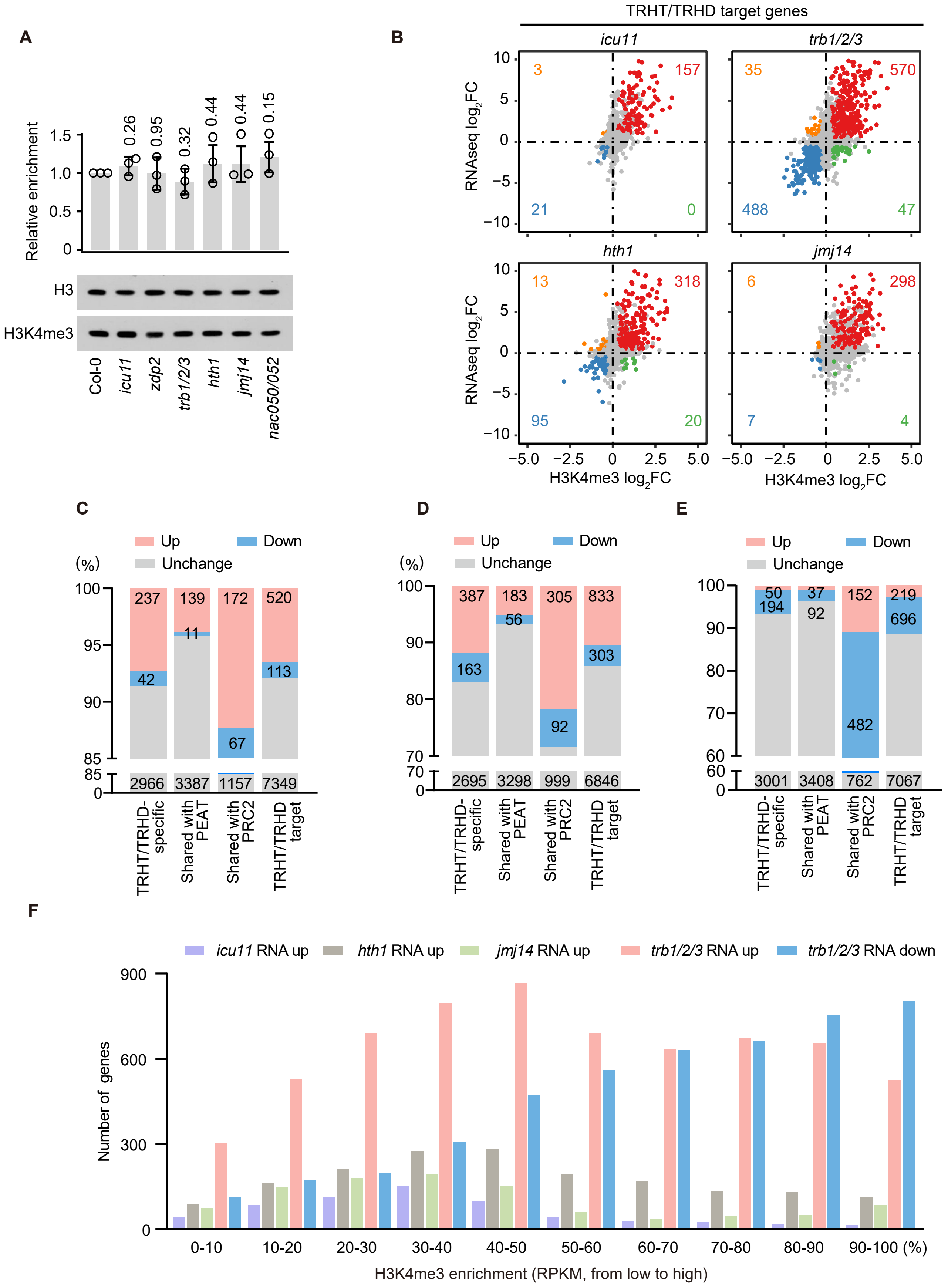

Supplemental Figure 9. Analysis of the effects of TRHT/TRHD mutations on H3K4me3 and gene expression.

(A) Determination of the effect of TRHT/TRHD mutations on the global H3K4me3 levels by western blot analysis. The histone H3 level served as a loading control. Quantifications of H3K4me3 signals are shown at the top of each panel. *P* values were determined by the two-tailed Student's t-test. Mean values and standard deviation (SD) are from three biological repeats.

(B) Scatter plots showing correlations between expression changes and H3K4me3 changes in *icu11, hth1, trb1/2/3*, and *jmj14* mutants compared to the wild type.

(C, D) Number of TRHT/TRHD target genes with up- and down-regulated H3K4me3 levels in the *icu11* (C) and *hth1* (D) mutants compared to the wild type. TRHT/TRHD target genes were classified into three subgroups for analysis: TRHT/TRHD-specific genes (3245), genes shared by PEAT (3537), and genes shared by PRC2 (1396). THRT/TRHD target genes refer to shared targets of TRHT and TRHD.

(E) Number of TRHT/TRHD target genes with up- and down-regulated H3K27me3 levels in the *trb1/2/3* compared to the wild type. TRHT/TRHD target genes were classified into the three subgroups described above.

(F) Graph illustrating the distribution of genes with expression changes in indicated mutants relative to the wild type across gene deciles. Total Arabidopsis genes (n = 32,548) were divided into deciles sorted by ascending levels of H3K4me3.

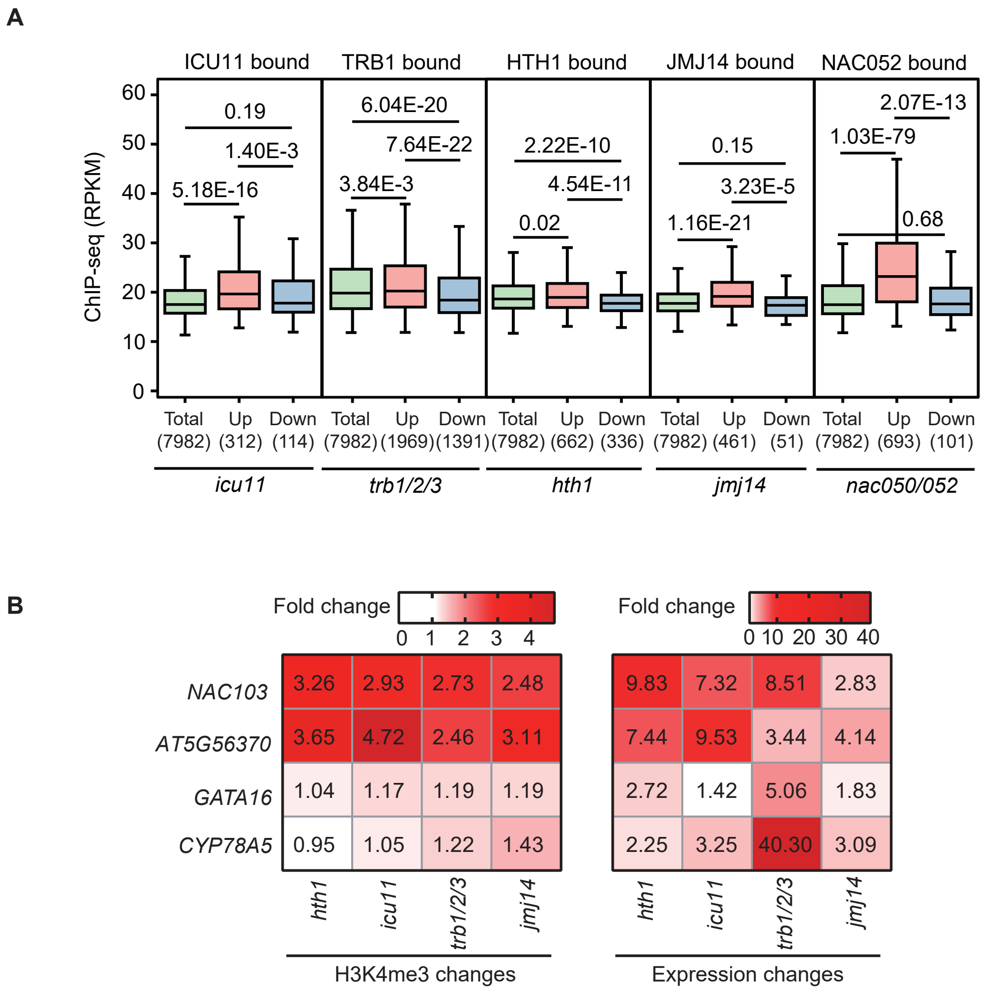

	hth1	icu11	trb1/2/3	jmj14
NAC103	3.26	2.93	2.73	2.48
AT5G56370	3.65	4.72	2.46	3.11
GATA16	1.04	1.17	1.19	1.19
CYP78A5	0.95	1.05	1.22	1.43

	hth1	icu11	trb1/2/3	jmj14
NAC103	9.83	7.32	8.51	2.83
AT5G56370	7.44	9.53	3.44	4.14
GATA16	2.72	1.42	5.06	1.83
CYP78A5	2.25	3.25	40.30	3.09

Supplemental Figure 10. TRHT/TRHD components mediate H3K4me3-independent transcriptional repression.
(A) ChIP-seq enrichment levels of TRHT/TRHD components at total TRHT/TRHD target genes and at those with up- or down-regulated expression. *P* values were determined by the Mann-Whitney U test (paired) for non-normally distributed data.
(B) H3K4me3 changes and expression changes of representative TRHT/TRHD target genes in mutants relative to the wild type. Color density indicates fold changes in H3K4me3 ChIP-seq enrichment (left) or RNA-seq enrichment (right) in mutants versus the wild type.

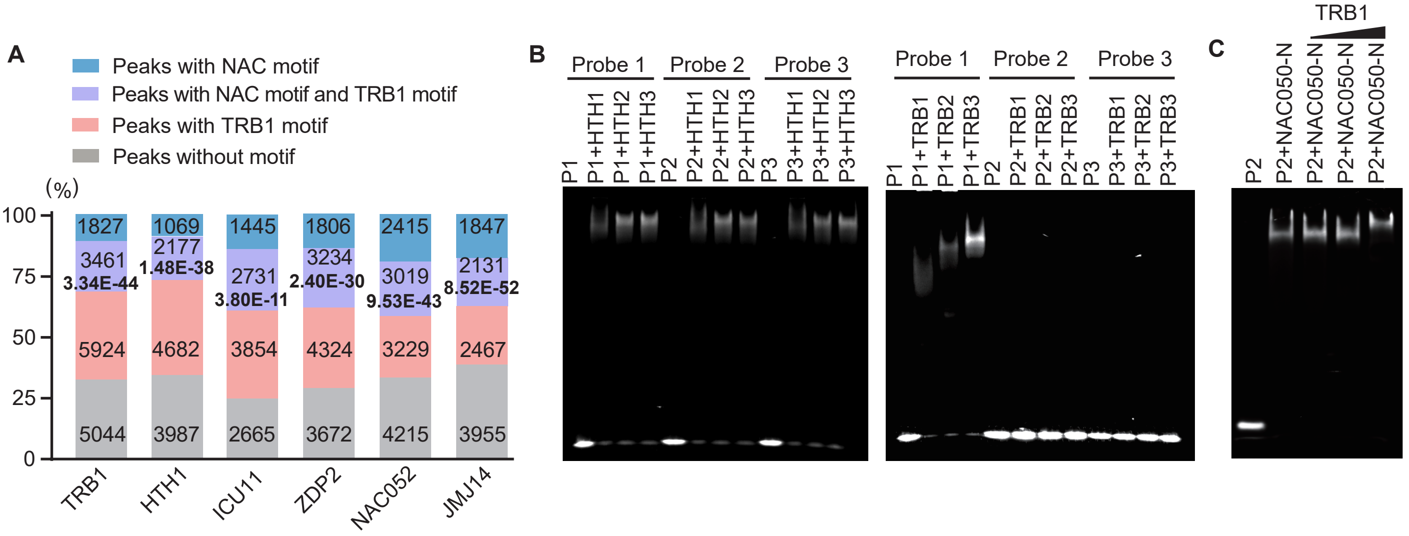

Supplemental Figure 11. Determination of DNA-binding abilities of TRB1/2/3 and HTH1/2/3 by EMSA.

(A) Bar plots showing the enrichment of the TRB1 motif and NAC motif in ChIP-seq peaks of the indicated TRHT/TRHD components. Statistical results are derived from HOMER. The significance of enrichment for each protein at peaks with both the TRB1 and NAC motifs was determined by calculating *P* values. *P* values were calculated using the one-tailed hypergeometric test and marked in bold black. The NAC motif refers to the motif enriched by NAC052 in ChIP-seq; the TRB1 motif refers to the motif enriched by TRB1 in ChIP-seq.

(B) Determination of the DNA-binding abilities of TRB1, TRB2, TRB3, HTH1, HTH2, and HTH3 by EMSA. Probe 1 (P1) and Probe 2 (P2) contain the TRB1 motif and NAC motif, respectively, while Probe 3 (P3) contains neither.

(C) Determination of the binding of NAC050-N to DNA in the presence or absence of TRB1. Probe 2 (P2) contains the NAC motif.

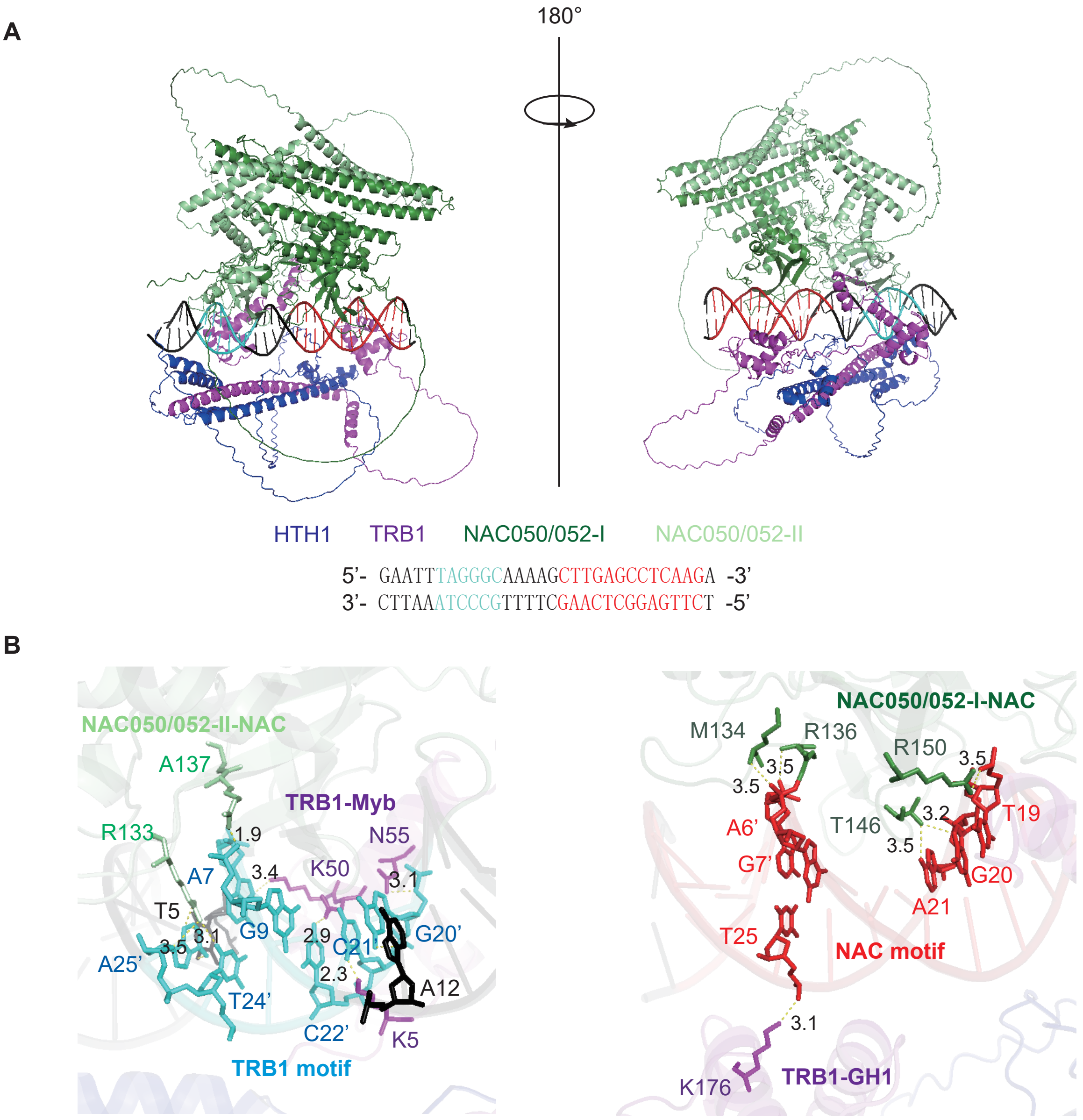

Supplemental Figure 12. Predicted DNA-binding sites of TRB1, NAC050, and HTH1 using the AlphaFold server.
(A) Predicted structure of the DNA-protein complex formed by NAC050/052, HTH1, and TRB1 bound to DNA. The structure model include two copies of NAC050/052 and one copy each of HTH1 and TRB1, interacting with DNA containing both TRB1 and NAC motifs. In the structure, NAC050/052-I, NAC050/052-II, TRB1, and HTH1 are shown in dark green, light green, purple, and blue, respectively. The TRB1 motif within the DNA is colored cyan, the NAC motif is shown in red, and all remaining DNA sequences are colored black.
(B) Predicted recognition of the TRB1 and NAC motifs by TRB1 and NAC050/052. The left panel shows recognition of the TRB1 motif by NAC050/052-II and TRB1. The involved bases are T5, A7, and G9 in the forward strand DNA, and G20', C21', C22', T24', and A25' in the reverse strand DNA. The right panel shows recognition of the NAC motif by NAC050/052-I and TRB1. The involved bases are T19, G20, A21, and T25 in the forward strand DNA, and A6' and G7' in the reverse strand DNA. Yellow dashed lines represent the interactions between amino acid residues and DNA, with numbers indicating distances (Å) and letters standing for the abbreviations of DNA-binding amino acids.

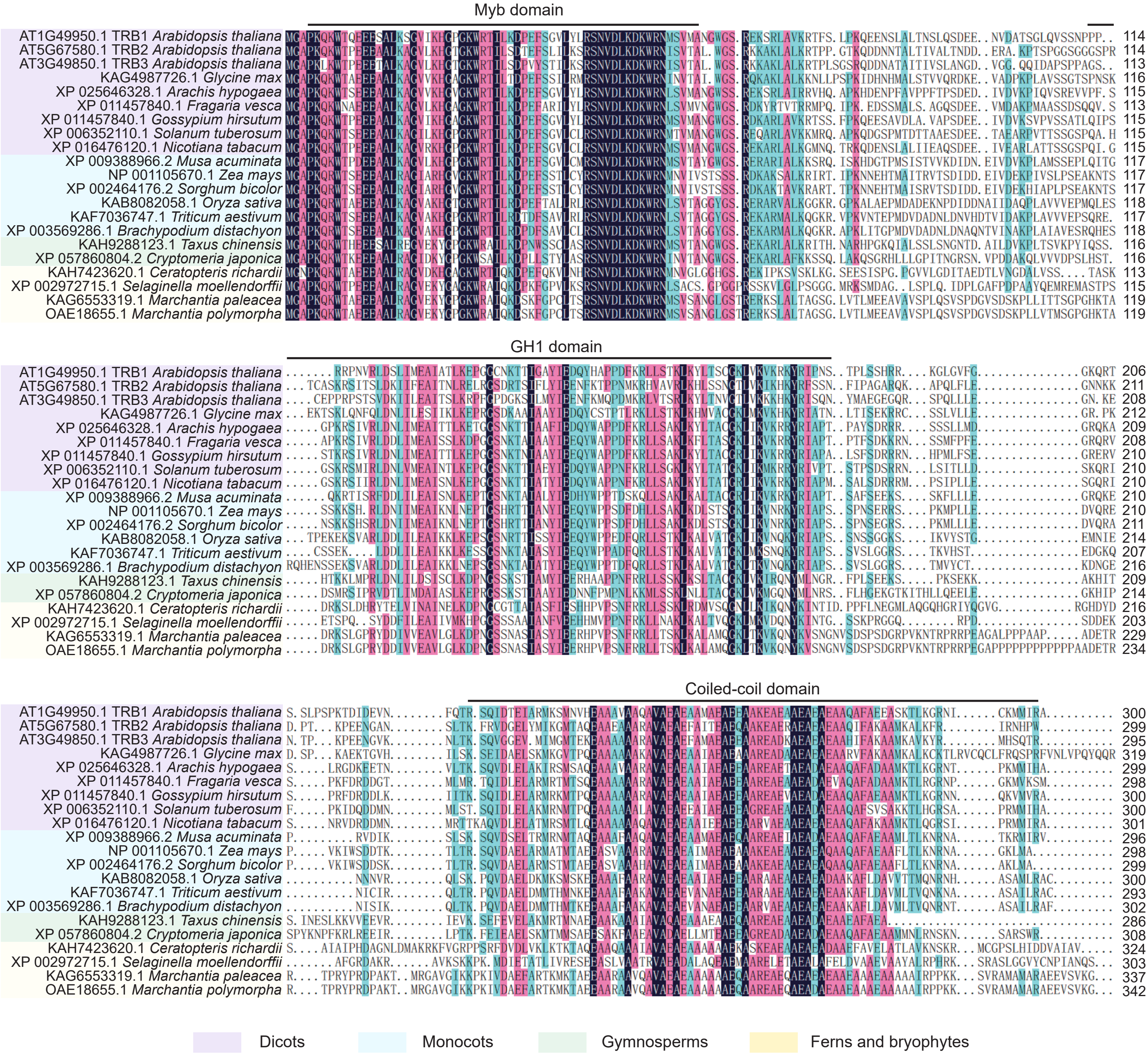

Supplemental Figure 13. Phylogenetic analysis of TRB1/2/3 homologs in different plant species.
TRB1/2/3 homologs in other species were identified via BLAST using Arabidopsis TRB1/2/3 as queries. Accession numbers are from the NCBI database. Conserved domains are highlighted.
